# A Multi-Scale Study of Water Dynamics under Confinement, Exploiting Numerical Simulations in Relation to NMR Relaxometry, PGSE and NMR Micro-Imaging Experiments: An Application to the Clay/Water Interface

**DOI:** 10.3390/ijms21134697

**Published:** 2020-06-30

**Authors:** Patrice Porion, Alfred Delville

**Affiliations:** Interfaces, Confinement, Matériaux et Nanostructures (ICMN), UMR 7374, CNRS and Université d’Orléans, 1b rue de la Férollerie, CS 40059, CEDEX 2, F-45071 Orléans, France

**Keywords:** diffusion in porous media, clay/water interface, nuclear magnetic resonance relaxometry, multi-quanta relaxometry, magnetic resonance imaging, pulsed-gradient spin-echo, two-time correlation function, self-diffusion tensor, confinement, multi-scale analysis, Brownian dynamics, molecular dynamics

## Abstract

Water mobility within the porous network of dense clay sediments was investigated over a broad dynamical range by using ^2^H nuclear magnetic resonance spectroscopy. Multi-quanta ^2^H NMR spectroscopy and relaxation measurements were first performed to identify the contributions of the various relaxation mechanisms monitoring the time evolution of the nuclear magnetisation of the confined heavy water. Secondly, multi-quanta spin-locking NMR relaxation measurements were then performed over a broad frequency domain, probing the mobility of the confined water molecules on a time-scale varying between microseconds and milliseconds. Thirdly, ^1^H NMR pulsed-gradient spin-echo attenuation experiments were performed to quantify water mobility on a time-scale limited by the NMR transverse relaxation time of the confined NMR probe, typically a few milliseconds. Fourthly, the long living quantum state of the magnetisation of quadrupolar nuclei was exploited to probe a two-time correlation function at a time-scale reaching one second. Finally, magnetic resonance imaging measurements allow probing the same dynamical process on time-scales varying between seconds and several hours. In that context, multi-scale modelling is required to interpret these NMR measurements and extract information on the influences of the structural properties of the porous network on the apparent mobility of the diffusing water molecules. That dual experimental and numerical approach appears generalizable to a large variety of porous networks, including zeolites, micelles and synthetic or biological membranes.

## 1. Introduction

In the last few decades, confined fluids were the subject of numerous experimental [1], theoretical [2] and numerical [3] studies because confinement strongly modifies the physico-chemical properties of bulk liquids. In that framework, the clay/water interface was frequently investigated [4,5,6,7,8,9,10,11,12] because clays exhibit a large variety of physico-chemical properties, such as water absorption, swelling, gelling, thixotropy, ionic exchange capacity and surface acidity exploited in numerous industrial applications, including waste storing [11], cosmetic and paint industries, drilling and heterogeneous catalysis [12]. Furthermore, clays are synthetic or natural materials, easily purified, with a well-controlled structure and atomic composition, leading to ideal models of solid/liquid interfaces. Optimising these numerous applications requires a detailed analysis of the influence of the clay composition on the structural and dynamical properties of the confined fluids.

**Figure 1:**
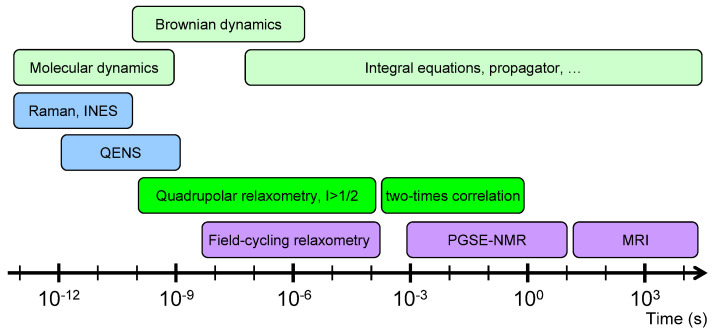
Schematic view of the different time-scales explored by various experimental and numerical dynamical studies.

In that context, a large number of experimental studies, including inelastic neutron scattering (INS) [13,14,15], quasi-elastic neutron scattering (QENS) [16,17], neutron spin echo [18], multi-quanta nuclear magnetic resonance (NMR) relaxometry (i.e., the frequency variation of the NMR relaxation rates) [19,20,21,22,23,24], NMR pulsed-gradient spin-echo (PGSE) attenuation [25,26,27,28,29,30] and NMR micro-imaging [29,30], were performed to quantify the mobility of confined water molecules over a broad range of diffusing time. The short times of mobility for the confined water molecules (from pico-seconds up to 100 nanoseconds) were easily probed by classical neutron scattering experiments (INS, QENS and neutron spin echo). Field-cycling NMR relaxometry is also a powerful tool to investigate the mobility of a large class of confined fluids [31,32,33,34,35,36,37,38,39,40] by probing the frequency variation of the relaxation rate. However, that method becomes useless when the NMR relaxation times of the confined fluids become smaller than the time required to switch the strength of the magnetic field. In that context, multi-quanta NMR relaxometry was successfully applied to probe water mobility in the intermediate time-scale (between microseconds and milliseconds). That time-scale may be extended up to seconds by measuring two-time correlation functions [22,23,24,41] of the long-living quantum state implied in the nuclear magnetisation of confined quadrupolar nuclei. Finally, NMR PGSE attenuation and NMR micro-imaging were also used to investigate the long-time water mobility (between seconds and hours). Because of the large time-scale covered by these numerous experiments (see Figure 1), multi-scale modelling is required to interpret and analyse these various experimental data by using quantum molecular dynamics (for INS) [15], classical molecular dynamics (for QENS and neutron spin echo) [17,18] or Brownian dynamics and differential equations [19,20,21,22,23,24,25,26,27,28,29,30,41] (for NMR relaxometry, two-time correlation function, PGSE and micro-imaging). The same approaches were applied to investigate the mobility of various cations (^7^Li [42], ^23^Na [43] and ^133^Cs [44]) neutralising the negative charge of the clay platelets. While this study focuses on the clay water interfaces, it may be successfully applied to other interfacial systems, including cementous material, zeolite, micro-porous silica [45,46,47,48], synthetic and natural macromolecules.

## 2. Materials and Methods

Three different classes of clay materials were used in that study: aqueous diluted dispersions [49], dense sediments [23] with a well-controlled hydration state and compacted aggregates [29]. While swelling clays (montmorillonie [50], beidellite [23]) are implied in the two first studies, the samples of the third study are prepared with kaolinite, i.e., unswelling clay [29]. The swelling clays result from the sandwiching of one octahedral layer of aluminum or magnesium oxides between two layers of tetrahedral silicon oxides. Because of some substitutions of these metallic atoms by less charged atoms, the individual clay platelet bears negative electric charge neutralised by solvated and exchangeable counterions. The charge locations and the nature of these counterions play central roles in the swelling behaviour of these clay platelets [50,51,52].

Montmorillonite was used to study dilute aqueous clay dispersions. It is a natural clay purchased from the Source Clays Mineral repository at Purdue University. It was prepared and purified according to classical procedure [50]. A sequence of centrifugation was also performed to reduce the size dispersion of the clay which is quantified by analysing TEM micrographs [49]. Two clay samples were selected for that study, with average lateral extents of (70±10) nm and (300±50) nm for the samples labelled Size3 and Size2 respectively. The general formula per unit cell of the purified montmorillonite is: (Al${}_{3.06}$Mg${}_{0.48}$Fe${}_{0.46}$)(Si${}_{7.76}$Al${}_{0.24}$)O${}_{20}$(OH)${}_{4}$Na${}_{0.77}$ [49].

**Figure 2:**
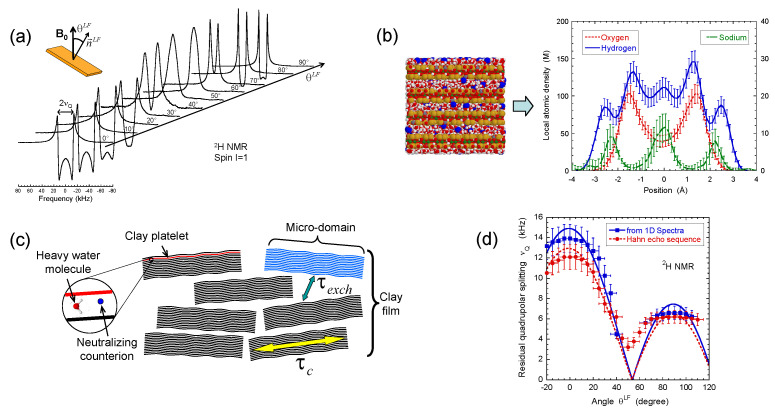
(**a**) ^2^H NMR spectra as a function of the film orientation $\theta^{LF}$ in the static magnetic field $B_{0}$; (**b**) Snapshot illustrating one grand canonical Monte Carlo (GCMC) equilibrium configuration of confined water molecules and neutralising sodium counterions from which one can deduce concentration profiles of sodium counterions with oxygen and hydrogen atoms pertaining to water molecules confined between two beidellite clay lamellae; (**c**) Schematic view of multi-scale organisation of the clay sediment resulting from the coexistence of clay aggregates with various orientations of the platelet directors; (**d**) Variation of the residual quadrupolar coupling $\nu_{Q}$ extracted from the ^2^H NMR spectra and the Hahn echo sequence as a function of the film orientation $\theta^{LF}$ in the static magnetic field $B_{0}$. Reprinted with permission from [23]. Copyright (2014) American Chemical Society.

Beidellite is natural clay used to prepare self-supporting clay films with well characterised degrees of hydration. It is purchased from the Source Clays Mineral repository at Purdue University and purified according the same classical procedures [50]. After purification, its general formula per unit cell is: (Al${}_{3.77}$Mg${}_{0.21}$Fe${}_{0.11}$)(Si${}_{7.27}$Al${}_{0.73}$)O${}_{20}$(OH)${}_{4}$Na${}_{0.67}$ [23]. Clay films are obtained by oedometric compression of diluted aqueous dispersions and dried under nitrogen flux before equilibration with a reservoir of heavy water (from Aldrich, purity 99.9%) at fixed water chemical potential by using saturated salt solutions [23]. We fix the water partial pressure *p*/$p_{0}$ = 0.92 by using saturated KNO${}_{3}$ aqueous solution of pure heavy water. This partial pressure was selected because it corresponds to a well characterised hydration state with an interlayer separation of 15.6 Å [53,54,55] corresponding to two hydration layers, as illustrated in Figure 2b. In order to avoid clay drying during the set of NMR experiments, a small reservoir of heavy water at fixed chemical potential is inserted in the cap of the NMR tube, out of the detection coil.

Kaolinite is the non-swelling clay selected for the last study. It results from the stacking of platelets composed from the superposition of one layer of tetrahedral silicon with one layer of octahedral aluminium oxides. The cohesion between individual platelets is insured by a dense network of hydrogen bonds between the hydroxides of octahedral aluminium oxides of one platelet and the oxygen atoms from the tetrahedral layer of silicon oxides pertaining to the neighbouring platelet. The kaolinite sample was purchased from the Source Clays Mineral repository at Purdue University. Its general formula per unit cell is: (Al${}_{1.9}$Ti${}_{0.065}$Fe${}_{0.035}$)(Si${}_{1.92}$Al${}_{0.08}$)O${}_{5}$(OH)${}_{4}$ [29,30]. Compacted clay samples are prepared by uniaxial compaction of the dry clay powder [29] and its porosity is determined by helium pycnometry [29].

**Figure 3:**
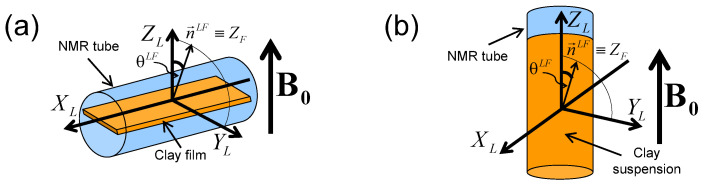
Schematic view of: (**a**) the clay film within the NMR tube inserted into the detection coil (see text) and (**b**) the clay suspensions into the NMR tube. The $X_{L}$ and $Z_{L}$ directors are parallel to the axes of the NMR detection coil and the static magnetic field $B_{0}$ respectively. The $\theta^{LF}$ Euler angle and the director ${\vec{n}}^{LF}$ characterise the orientation of the clay sample by reference to the static magnetic field $B_{0}$. Reprinted with permission from [23]. Copyright (2014) American Chemical Society.

^2^H NMR spectra and relaxation measurements were performed on a DSX360 Bruker spectrometer operating at a field of 8.465 Tesla. On this spectrometer, typical pulse duration for the total inversion of the magnetisation is equal to 23 microseconds. Spectra were recorded by using a fast acquisition mode with a typical sampling time of 0.25 microseconds, corresponding to a spectral width of 4 MHz. The NMR spectra are classically recorded by using a single detection pulse. Rectangular clay lamellae (typically 30×5.5 mm${}^{2}$) are cut in the self-supporting clay films and are inserted in a sealed cylindrical tube (see Figure 3a) fitting the gap of the home-made solenoidal coil used to perform the various NMR measurements [20,21,22,23,24,27,28,29,30,41,42,43,44]. As a consequence, these NMR measurements can be performed at different orientations of the clay film by reference with the static magnetic field $B_{0}$ (see Figure 3a). In the case of aqueous clay dispersions, this setup was slightly modified: the sample holder was a tube fitting the diameter of the coil, and its axis was oriented perpendicularly to the coil axis (see Figure 3b). It can be rotated by 90${}^{{}^{\circ}}$ while maintaining the sample in the magnet, allowing one to perform dynamical studies of the clay mobility into the static magnetic field $B_{0}$.

^1^H NMR PGSE self-diffusion and magnetic resonance imaging (MRI) measurements are performed on a Bruker DSX100 spectrometer operating at a static magnetic field $B_{0}$ of 2.351 Tesla. This spectrometer is equipped with a saddle coil, with the axis of the sample holder parallel to the static magnetic field $B_{0}$. Typical pulse duration for a complete inversion of the proton magnetisation is 10 microseconds.

## 3. Results and Discussion

### 3.1. NMR Spectra

**Figure 4:**
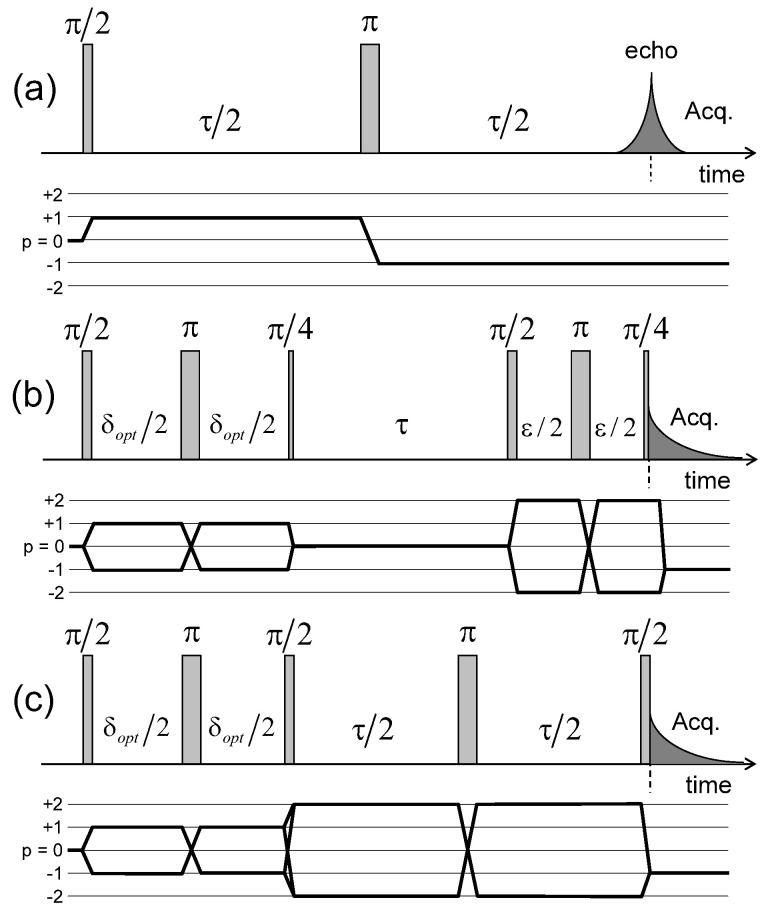
Pulse sequences and coherence transfer pathways used to measure the NMR relaxation rates of: (**a**) $T_{11}\left(a,s\right)$; (**b**) $T_{20}$ and (**c**) $T_{22}\left(a,s\right)$ coherences (see the Appendix C). The delay $\delta_{opt}$ is selected to optimise the coherence tranfer, and the delay ϵ is set equal to 10 μs. Reprinted with permission from [23]. Copyright (2014) American Chemical Society.

In contrast with bulk water, splitting of the ^2^H NMR resonance line of heavy water (see Figure 2a) is a general trend of the confined water molecules in the presence of clay platelets [56,57,58]. It results from a partial average of the instantaneous quadrupolar coupling (see Equation (A5) in the Appendix A) felt by the deuterium atom. Since its principal axis is oriented along the $\vec{OD}$ director [59], any asymmetry of the water reorientation motions induces such residual quadrupolar coupling. The NMR signal of bulk water in the outer reservoir (see Section 2) induces the distortion of the NMR spectra (see Figure 2a). Therefore, the data displayed in Figure 2d were compared [23] with the residual quadrupolar coupling, noted $\nu_{Q}$, extracted from the oscillations of the transverse magnetisation detected by using a Hahn echo pulse sequence (see Figure 4a). As displayed in Figure 2d, the agreement between both set of measurements is quantitative. As predicted by molecular modelling of the clay/water interface [54,55,60], water molecules exhibit specific orientation in the vicinity of the clay surface. As illustrated in Figure 2b, the configurations of the water molecules obtained by grand canonical Monte Carlo simulations confirm the specific orientation of confined water molecules in contact with the clay surface (see Figure 6c in the reference [23]) and are responsible for the splitting of the ^2^H NMR spectra. As a consequence, the observed residual splitting results from an average over the distribution of the clay directors (see Equation (A6) in the Appendix A) inside the clay film in addition to the specific organisation of the water molecules confined in the interlamellar space of the clay platelets (see Figure 2c), leading to an apparent residual splitting varying according to the film orientation inside the static magnetic field $B_{0}$ (see Figure 2d).

**Figure 5:**
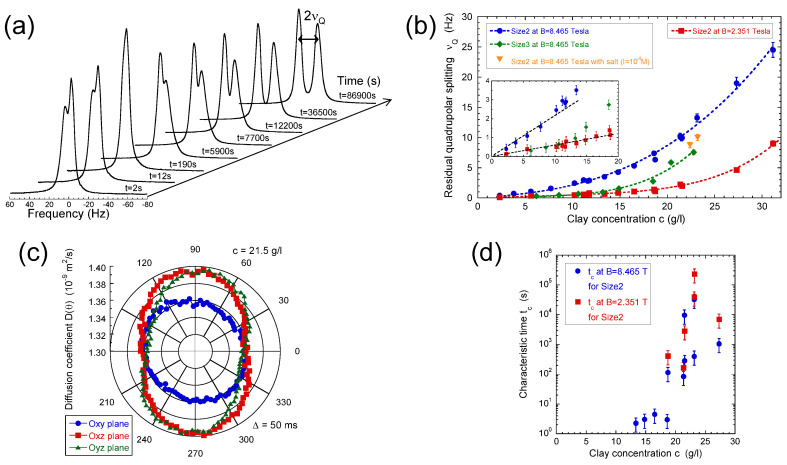
(**a**) ^2^H NMR spectra recorded during the recovery of the residual quadrupolar splitting $\nu_{Q}$ after a 90${}^{{}^{\circ}}$ rotation of a previously equilibrated clay sample; (**b**) Concentration variations of the limiting equilibrium residual quadrupolar splitting $\nu_{Q}$ measured at $B_{0}$ = 2.351 and 8.465 Tesla for the Size2 clay samples and at $B_{0}$ = 8.465 Tesla for the Size3 clay samples. Additional measurements are performed at $B_{0}$ = 8.465 Tesla for the Size2 samples in the presence of salt (*I* = 10${}^{-4}$ M); (**c**) Determination of the various components of self-diffusion tensor D of the water molecules in the Size2 clay sample (c = 21.5 g/L) measured by the ^2^H NMR PGSE method at $B_{0}$ = 2.351 Tesla. The PGSE measurements are performed after equilibration of the clay platelet orientation in the static magnetic field $B_{0}$ parallel to the ${\vec{e}}_{z}$ director; (**d**) Concentration variation of the characteristic time $t_{c}$ measured at $B_{0}$ = 2.351 and 8.465 Tesla for the Size2 clay samples. Reprinted with permission from [49]. Copyright (2010) American Chemical Society.

Such residual quadrupolar coupling was also detected (Figure 5a,b) in the presence of dilute and isotropic aqueous clay dispersions [49,56,57,58] resulting from the individual platelet orientation induced by the static magnetic field $B_{0}$. Such a platelet alignment in the static magnetic field results from the large anisotropy of their magnetic susceptibility [49]. As previously illustrated [25], the water self-diffusion tensor may be used to identify the preferential orientation of the clay directors. As shown in Figure 5c, the clay directors of dilute aqueous dispersions of montmorillonite are preferentially oriented perpendicular to the static magnetic field. Figure 5a also exhibits reduced asymmetry of the water doublet. It results from the paramagnetic impurities (iron) of the clay platelets. As detailed in the Appendix, the resulting paramagnetic coupling interferes with the quadrupolar coupling responsible for the deuterium relaxation and induces such asymmetry of the deuterium doublet [57,61]. As displayed in Figure 5a, the platelet orientation is not instantaneous and depends upon the clay concentration and the strength of the static magnetic field (see Figure 5b). The strong enhancement of the time-scale characterising the clay reorientation in the static magnetic field reported in Figure 5d is the fingerprint of their mutual repulsion resulting from the overlap of their ionic diffuse layers of neutralising counterions [1,49,61]. It corresponds to a microscopic detection of the sol–gel transition occurring at slightly lower clay concentrations than those detected by macroscopic rheological measurements [49].

### 3.2. NMR Relaxometry

The second source of difference between isotropic and confined liquids is given by the orders of magnitude of the longitudinal ($R_{1}$ = $1/T_{1}$) and transverse ($R_{2}$ = $1/T_{2}$) NMR relaxation rates: while both relaxation rates are general equal in bulk liquids at room temperature, confinement induces a large enhancement of the NMR transverse relaxation rate by reference to its longitudinal counterpart (see Appendix). In the framework of the classical BPP model [61,62], this behaviour was previously assigned [57,63] to a large increase of the time-scale characterising the decorrelation of the relaxation mechanism monitoring the NMR relaxation process of the confined molecules by reference to their dynamical behaviour in bulk liquid. While the classical BPP model adequately interprets the NMR relaxation process in bulk liquids, it becomes useless in analysing NMR relaxation of the same confined fluid. As an example, the ^2^H NMR relaxation rate of bulk heavy water ($R_{2}$ = $R_{1}$ = 2.4 s${}^{-1}$) is compatible with an orientational correlation time of a few pico-seconds, in agreement with QENS measurements. By contrast, the large enhancement of ^2^H transverse relaxation time of heavy water in the presence of swelling clays ($R_{2}$ = 13,000 s${}^{-1}$ and $R_{1}$ = 110 s${}^{-1}$) [64] requires, in the framework of the BPP model, an orientational correlation time of 20 nanoseconds while its order of magnitude reported by QENS measurements still fits a few pico-seconds! As a consequence, one must definitively reject any interpretation of the NMR relaxation rates of confined fluids on the basis of this classical BPP model. In that context, numerous analytical [36] and numerical [65,66,67] models were developed to interpret such enhancement of the NMR transverse relaxation detected for confined fluids. In contrast with the isotropic BPP model, that interpretation of the NMR relaxation of confined fluids focuses on the diffusion time [36,65,66,67] required by the confined molecular probe to explore different local environments and lose the memory of their initial configuration.

In the case of dilute aqueous dispersions [19,49,57], that diffusion time corresponds to the average residence time $\tau_{c}$ of the water molecules at the surface of the clay particle. For dense clay sediments, the same diffusion time corresponds to the average residence time $\tau_{c}$ of the water molecules confined between two clay platelets [20,21,22,23,24]. NMR relaxometry measurements are then performed to investigate the frequency variation of the NMR relaxation rates and extract the average residence time $\tau_{c}$ of the diffusing probes. The order of magnitude of the residence time $\tau_{c}$ is simply evaluated by inverting the characteristic angular velocity $\omega_{c}$. In that context, determining the average size of these clay platelets is a simple way to estimate the order of magnitude of the mobility of these confined water molecules [20,21,22,23,24].

One may be tempted to interpret the large enhancement of the transverse relaxation rate on the basis of iron clusters dispersed in the clay sediments and inducing heterogeneities of the magnetic susceptibility within the mineral. This analysis is not valid in our case since the longitudinal relaxation rate, a priori not affected by heterogeneities of the chemical shift [68]), is also enhanced compared to its value in the bulk. To avoid such heterogeneities of the magnetic susceptibility, we used either synthetic or purified natural clays (see Section 2) in which iron atoms are only present in limited quantity and dispersed within the clay matrix by substituting metallic atoms of the clay network (see Section 2). Furthermore, the paramagnetic contribution of these atoms to the relaxation mechanisms is fully described in our analysis (see Appendix A and Appendix D) and the multi-quanta relaxation measurements were performed to separately quantify the contributions of the quadrupolar and paramagnetic relaxation mechanisms. Nevertheless, we also observed large enhancement of transverse relaxation rate of the confined water molecules in comparison with their longitudinal relaxation rate, resulting from the slow modulation of these relaxation mechanisms. This was previously demonstrated by measuring, at various strengths of the static magnetic field, the relaxation rates of confined quadrupolar nuclei in the presence of synthetic and natural but purified clay sediments [69]. While the *transverse* relaxation rate appears *independent* of the static magnetic field $B_{0}$, the *longitudinal* relaxation rate *increases* with a *decrease* of the magnetic field. A completely opposite trend should be detected for relaxation enhancements induced by the heterogeneities of the magnetic field; i.e., *no influence* of the strength of the static magnetic field on the *longitudinal* relaxation rates and an *increase* of the *transverse* relaxation rate as a function of the *increase* of the static magnetic field!

The relaxation of the deuterium nuclei is mainly monitored by the quadrupolar coupling. As explained in the Appendix (see Appendix A), this quadrupolar coupling is modulated only by the reorientation of the water molecule (see Equations (A4) and (A5)), more precisely by the fluctuations of the $\vec{OD}$ director into the static magnetic field $B_{0}$, since the principal axis of the electrostatic coupling felt by the deuterium atom of heavy water is oriented along the $\vec{OD}$ director [59]. This is the reason why we should expect a short correlation time (i.e., a few ps), in agreement with QENS measurements [17]. This situation differs strongly from the modulation of the dipolar coupling, which includes also a contribution from the separation between the pair in interacting dipolar nuclei; see Equation (A9). As a consequence, molecular diffusion of the confined dipolar probes becomes an important source of NMR relaxation, inducing a slow modulation of the NMR dipolar coupling in the case of confined fluids [33,35,36,39,67,70]. However, because of the specific orientation of the water molecules confined in the interlamellar space between two clay platelets, the orientation fluctuations of the $\vec{OD}$ director of these confined water molecules is strongly restricted until their desorption. This point is well illustrated by numerical simulations [21]. As a consequence, the residence time of the water molecules adsorbed within in clay porous network monitors the decorrelation of their quadrupolar coupling, inducing the abovementioned tremendous increase of the correlation time.

**Figure 6:**
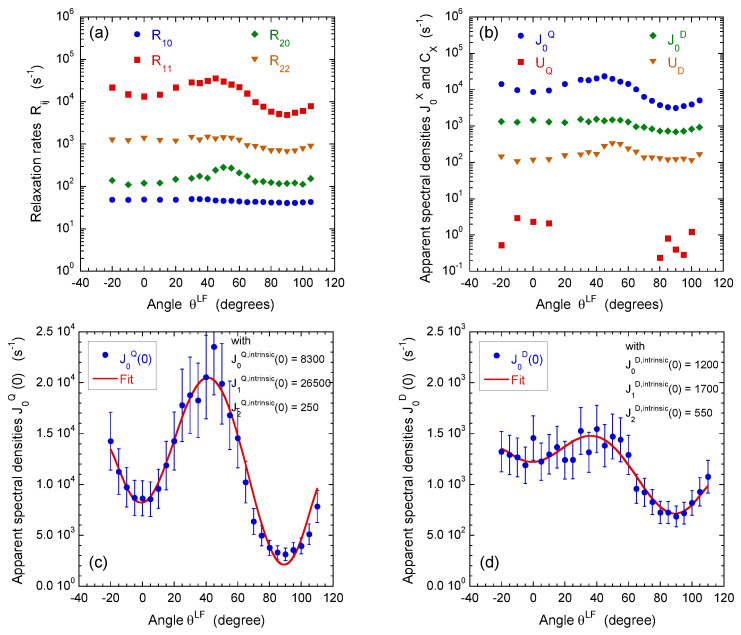
Influence of film orientation $\theta^{LF}$ in the static magnetic field $B_{0}$: (**a**) on the apparent multi-quanta relaxation rates of $T_{10}$, $T_{11}\left(a,s\right)$, $T_{20}$ and $T_{22}\left(a,s\right)$ coherences, noted $R_{10}$, $R_{11}$, $R_{20}$ and $R_{22}$, respectively; and (**b**) on the on the apparent spectral densities $J_{0}^{Q}\left(0\right)$, $J_{0}^{D}\left(0\right)$, $U_{Q}$ and $U_{D}$ extracted from these $R_{ij}$ values; see Equation (A24) in Appendix D. The intrinsic spectral densities $J_{0}^{X,intr}\left(0\right)$, $J_{1}^{X,intr}\left(0\right)$ and $J_{2}^{X,intr}\left(0\right)$ with X∈{D,Q}, extracted from angular variation of the apparent spectral densities $J_{0}^{X}\left(0\right)$ quantifying (**c**) heterogeneous quadripolar (X=Q) and (**d**) dipolar (X=D) relaxation mechanisms (see the Appendix). Reprinted with permission from [23]. Copyright (2014) American Chemical Society.

For that purpose, a set of multi-quanta NMR relaxation measurements are performed with the pulse sequences illustrated in Figure 4a–c. Figure 6a displays the corresponding relaxation rates from which we can extract the apparent spectral densities measured in the laboratory frame (Figure 6b); see Equations (A21) and (A22a)–(A22e) in the Appendix D. In that approach, we focus on the dominant contributions to the NMR relaxation mechanisms. As detailed in the Appendix B in Equations (A10)–(A15), the so-called spectral densities J(ω) are used to evaluate the contributions of the various relaxation mechanisms to the NMR relaxation rates. Since under confinement each spectral density (see Equation (A23)) evaluated at zero angular velocity is always larger than its counterpart evaluated at high angular velocity,

(1) $$J\left(0\right)\;\gg \;J\left(\omega_{0}\right)\;\geq \;J\left(2\omega_{0}\right)$$

we obtain a limited number of parameters characterising all the multi-quanta NMR relaxation rates (see Equation (A24) in the Appendix D). By using Wigner rotation matrices (cf. Equations (A16a)–(A16c) in the Appendix B), we can easily extract from these apparent spectral densities their intrinsic counterparts evaluated in the frame of the oriented clay film in which the water diffusional process occurs. As shown in the Appendix D in the Equation (A24), a limited number of parameters are required to fully quantify the angular variation of the multi-quanta relaxation rates. That analysis allows not only distinguishing the relative contributions from the quadrupolar and paramagnetic relaxation mechanisms but also determines the intrinsic spectral densities evaluated at zero angular velocity in the frame of the clay film (see Figure 6c,d and Equations (A16a)–(A16c) in the Appendix B). As illustrated in Figure 6c, the apparent spectral density of the quadrupolar coupling $J_{0}^{Q}\left(0\right)$ exhibits a large angular variation because its intrinsic contributions significantly differ

(2) $$J_{1}^{Q,intrinsic}\left(0\right)\;\gg \;J_{0}^{Q,intrinsic}\left(0\right)\;>\;J_{2}^{Q,intrinsic}\left(0\right)$$

while the angular variation of the apparent spectral density of the paramagnetic coupling ($J_{0}^{D}\left(0\right)$) is less noticeable (Figure 6d) because of the limited difference between its intrinsic components

(3) $$J_{0}^{D,intrinsic}\left(0\right)\;≃\;J_{1}^{D,intrinsic}\left(0\right)\;>\;J_{2}^{D,intrinsic}\left(0\right)$$

These relationships are not specific to water molecules confined within beidellite sediments but were also reported for various clay sediments [20,21,22,23,24].

**Figure 7:**
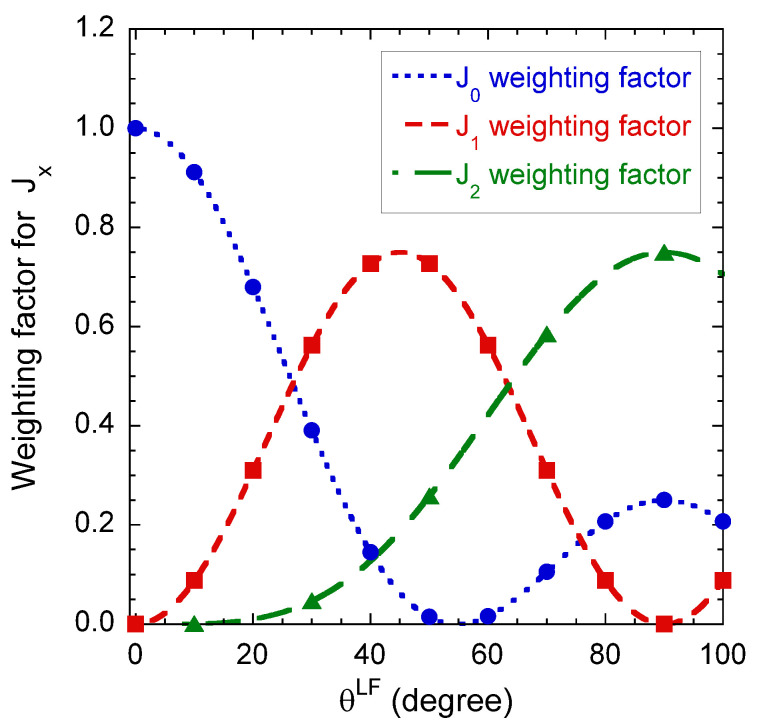
Angular variations of the relative weights of the various intrinsic contributions $J_{m}^{X,intr}\left(\omega \right)$ to the m=0 component of the apparent spectral density $J_{0}^{X}\left(\omega \right)$ monitoring the quadrupolar and dipolar relaxation mechanisms (see Equation (A16a) in the Appendix B).

Figure 7 illustrates the angular variation of the relative weight of the various intrinsic contributions to the m=0 component of the apparent spectral density monitoring the quadrupolar and dipolar relaxation mechanisms (see Equation (A16a) in the Appendix B). By coupling these results with the data displayed in Figure 6c,d, it becomes possible to extract all the required dynamical information by performing a limited number of multi-quanta relaxometry measurements. For that purpose, we selected only three different orientations of the clay film into the static magnetic field; i.e., 0${}^{{}^{\circ}}$, 90${}^{{}^{\circ}}$ and 30${}^{{}^{\circ}}$. At 0${}^{{}^{\circ}}$, only the m=0 intrinsic component contributes to the apparent relaxation process. Then, measurements performed at 90${}^{{}^{\circ}}$, still remove the contribution from the m=1 intrinsic component, but simultaneously optimise the contribution of the m=2 intrinsic component. Finally, an intermediate angle (30${}^{{}^{\circ}}$) is selected to add a reduced contribution of the m=1 intrinsic component. We avoid using the angle value 45${}^{{}^{\circ}}$, i.e., the angle optimising this contribution, because of the large enhancement expected to occur from the quadrupolar relaxation mechanism (see Figure 6c).

**Figure 8:**
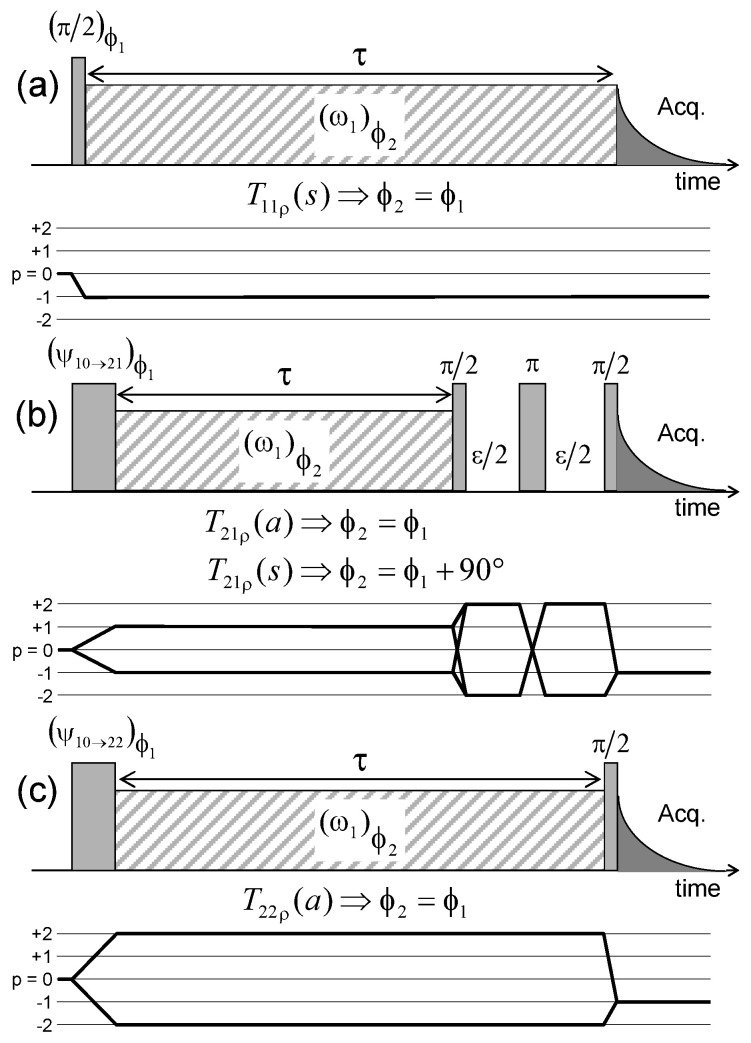
Pulse sequences and coherences transfer pathways used to measure the multi-quanta spin-locking relaxation rates of the (**a**) $T_{11}\left(s\right)$; (**b**) $T_{21}\left(a\right)$ and $T_{21}\left(s\right)$; and (**c**) $T_{22}\left(a\right)$ coherences, noted $T_{11\rho }\left(s\right)$, $T_{21\rho }\left(a\right)$, $T_{21\rho }\left(s\right)$ and $T_{22\rho }\left(a\right)$ respectively. The delay ϵ is set equal to 10 μs. The pulse durations $\psi_{10\to 21}$ and $\psi_{10\to 22}$ are selected to optimise magnetisation transfer between the corresponding coherences; i.e., $T_{10}\to T_{21}$ and $T_{10}\to T_{22}$ respectively. Reprinted with permission from [23]. Copyright (2014) American Chemical Society.

**Figure 9:**
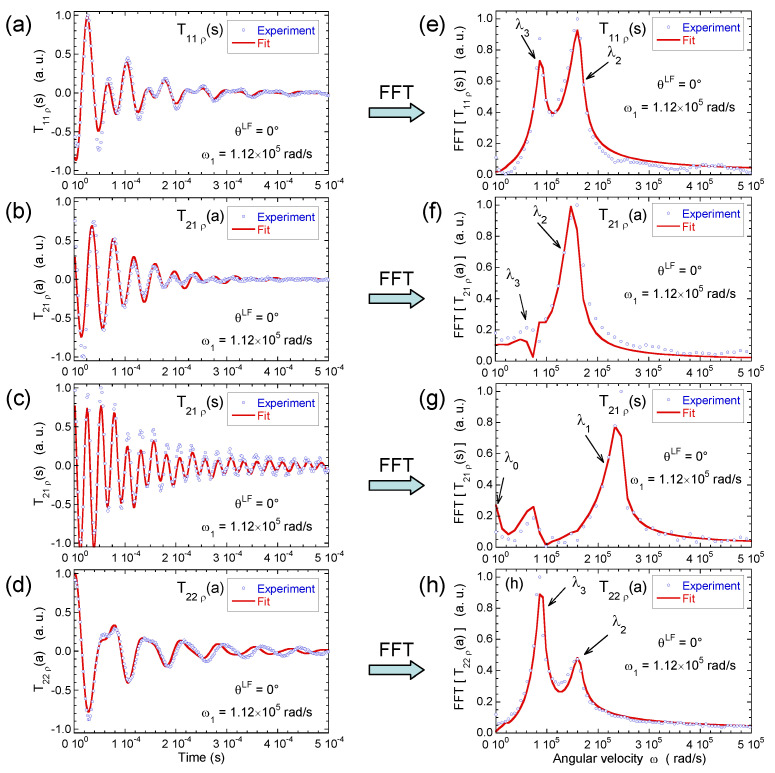
Time evolution of (**a**) $T_{11}\left(s\right)$; (**b**) $T_{21}\left(a\right)$; (**c**) $T_{21}\left(s\right)$ and (**d**) $T_{22}\left(a\right)$ coherences measured under spin-locking conditions, noted $T_{11\rho }\left(s\right)$, $T_{21\rho }\left(a\right)$, $T_{21\rho }\left(s\right)$ and $T_{22\rho }\left(a\right)$, respectively; and their Fourier transforms for (**e**) $T_{11}\left(s\right)$; (**f**) $T_{21}\left(a\right)$; (**g**) $T_{21}\left(s\right)$ and (**h**) $T_{22}\left(a\right)$ coherences (^2^H NMR). Reprinted with permission from [23]. Copyright (2014) American Chemical Society.

**Figure 10:**
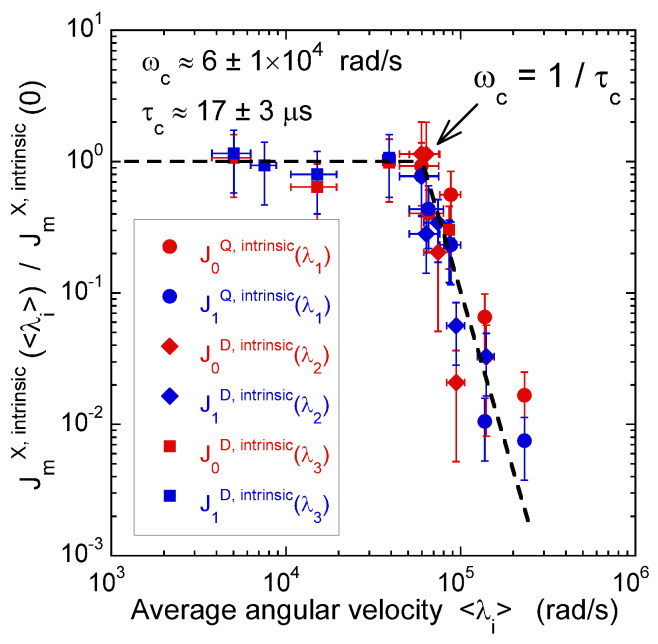
Variation of the intrinsic spectral densities $J_{m}^{Q,intr}\left(\lambda_{1}\right)$ with m∈{0,1}, describing quadrupolar coupling, and $J_{m}^{D,intr}\left(\lambda_{i}\right)$ with m∈{0,1} and i∈{2,3}, describing the heteronuclear dipolar coupling, as a function of the corresponding averaged angular velocities $<\lambda_{i}>$ (^2^H NMR). Reprinted with permission from [23]. Copyright (2014) American Chemical Society.

Figure 8 illustrates the pulse sequence used for these multi-quanta NMR relaxometry measurements. Relaxometry measurements were already performed for various confined fluids [19] by using only NMR locking of the longitudinal and transverse magnetisations of dipolar nuclei (spin I = 1/2). The use of quadrupolar nuclei (spin I > 1/2) allows performing here multi-quanta relaxometry, increasing significantly the range of probed angular velocities. This point is well illustrated In Table 1 by listing the broad range of probed angular velocities with a limited number of irradiation powers corresponding to the values of $\omega_{1}$. Figure 9 displays the typical agreement between experimental data and fitted curves. Fourier transforms of these results better illustrate the specific resonance frequencies (see Equation (A20) in the Appendix) probed by these multi-quanta spin-locking relaxation measurements. Finally, Figure 10 displays the variation of the reduced spectral densities probed by these multi-quanta NMR experiments. Two frequency domains are clearly displayed, with a plateau at low frequency and a unique monotonous decrease at high frequency. The transition between these two dynamical regimes occurs at $\left(6\pm 1\right)\times 10^{4}$ rad/s, leading to a characteristic residence time of (17±3) microseconds for the water molecules confined within the interlamellar space of beidellite clay, in agreement with the data obtained by QENS measurements [17] and MD numerical simulations [17] performed at a much smaller time-scale. Such agreement was also obtained for various heavy water molecules confined between clay lamellae under controlled hydration conditions [20,21,22,24].

### 3.3. Two-Time Correlation Function

**Figure 11:**
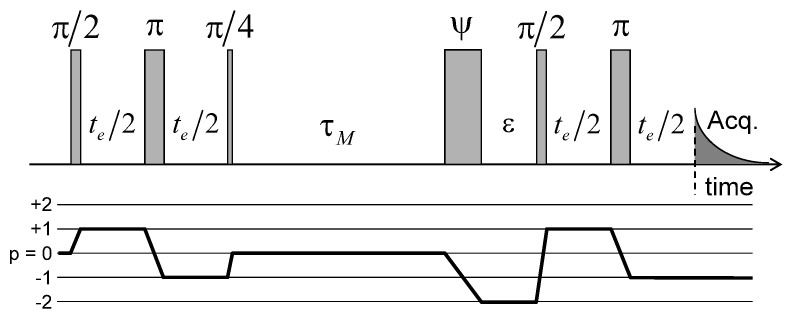
Pulse sequence and coherence pathway used to measure the attenuation of the two-time ^2^H NMR stimulated echo $I(t_{e},\tau_{M})$ as a function of the evolution period $t_{e}$ and the mixing time $\tau_{M}$. The fourth pulse duration, noted ψ, is optimised in order to maximise the transfer from $T_{20}$ to $T_{22}\left(s\right)$ coherence and simultaneously minimise the transfer from $T_{10}$ to $T_{22}\left(a\right)$ coherence. Reprinted with permission from [23]. Copyright (2014) American Chemical Society.

Dynamical information on the long-time mobility of the confined water molecules may be extracted by measuring two-time correlation function. The pulse sequence used to perform said measurement is displayed in Figure 11: it is based on echo attenuation measurement by saving the magnetisation during the long evolution period ($\tau_{M}$) on the long-living $T_{20}$ coherence or quantum spin state (cf. Equations (A21)–(A24) in the Appendix). That sequence exploits the heterogeneity of the platelet orientations within the clay sediment (see Figure 2c). During the first evolution period, the first-order coherence $T_{10}$ oscillates according to the initial local value of the residual quadrupolar coupling (noted $\omega_{Q}\left(0\right)$) felt by the confined water molecules. After a mixing period $\tau_{M}$, the same water molecules diffuse through the clay film and may reach a new clay aggregate with a different orientation within the film, corresponding to another residual quadrupolar coupling (noted $\omega_{Q}\left(\tau_{M}\right)$)). As a consequence, the measured magnetisation evolves according to [41,71,72]:

(4) $$I\left(t_{e},\tau_{M}\right)=\langle \cos\left(\omega_{Qi}\left(0\right)t_{e})\right)\times \cos\left(\omega_{Qj}\left(\tau_{M}\right)t_{e})\right)\rangle \times e^{-(R_{20}\tau_{M}+2R_{11}t_{e})}$$

If the mixing time $\tau_{M}$ is shorter than the time-scale characterising water exchange between platelets pertaining to different orientations, both residual quadrupolar couplings are the same and Equation (4) predicts damped oscillations of a squared cosinus function. By contrast, a strong attenuation of these oscillations is expected to occur if the mixing time exceeds the residence time of the water molecules confined within their initial aggregate.

**Figure 12:**
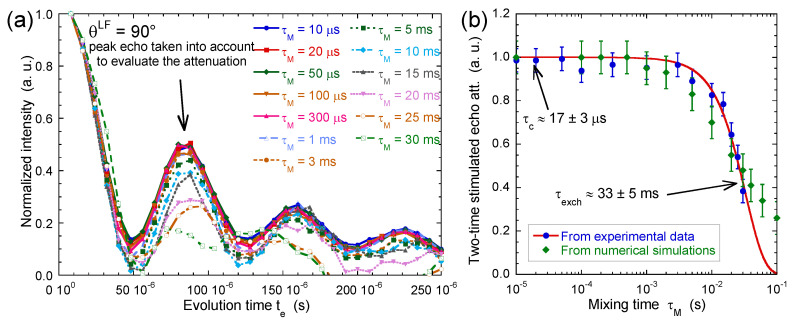
(**a**) Variation of the two-time stimulated echo attenuation $I(t_{e},\tau_{M})$ as a function of mixing time $\tau_{M}$ (^2^H NMR). The data are normalised to take into account the relaxation of the $T_{20}$ coherence during the mixing time $\tau_{M}$ (see Equation (4)); (**b**) Two-time correlation function extracted from the normalised stimulated echo attenuation as a function of the mixing time $\tau_{M}$. The red line corresponds to the best fit of a stretched exponential function, $f\left(t\right)=A\exp{\left(-t/\tau_{exch}\right)}^{\alpha }$, to determine the exchange time $\tau_{exch}$ ($\tau_{exch}=33\pm 5$ ms with an exponent α set equal to 1.5). The green line dots are obtained by numerical modelling (see text). Reprinted with permission from [23]. Copyright (2014) American Chemical Society.

Figure 12a results from measurements performed on the same beidellite clay sample. After normalisation of the first point, Figure 12b displays a noticeable decrease of the intensity of the abovementioned oscillations of the initial squared cosinus function resulting from water exchange between aggregates with different orientations within the clay film. The exponent α = 1.5 is heuristic and has no physical meaning. It results from a fit of both experimental and numerical data and is used to quantify the order of magnitude of the time $\tau_{exch}$. The time-scale corresponding to said exchange $\tau_{exch}$ is quite long (33±5) milliseconds, much longer than the average residence time $\tau_{c}$ of the same water molecules in the interlamellar space inside the same aggregate (17±3) microseconds. By using the self-diffusion coefficient of bulk water $D_{bulk}$, one can estimate the upper limit of the correlation length characterising the propagation of the orientation ordering of the clay aggregates within the film

(5) $$L_{max}=\sqrt{2\;D_{bulk}\;\tau_{exch}}=11\pm 2\;\mu m$$

that is one order of magnitude larger than the average size of the beidellite platelets used in that study.

The decrease of the two-time correlation function displayed in Figure 12b is modelled by a set of differential equations describing the exchange between 3375 elementary cubic sub-cells within the clay sediment, each characterised by its residual quadrupolar coupling [41]. A Gaussian random field, with a priori field-field correlation [73,74], was used to generate the distribution of the 3375 local values of the residual quadrupolar coupling. The standard deviation of this Gaussian field–field correlation function [41] was selected to match the length of two elementary sub-cells. Each sub-cell is indexed by three indices (i,j,k), allowing us to describe water exchange between the magnetisation pertaining to neighbouring sub-cells:

(6) $$\frac{d\sigma_{i,j,k}}{dt}=\left(R_{i,j,k}-6k_{exch}t\right)\sigma_{i,j,k}+k_{exch}t\left[\sigma_{i+1,j,k}+\sigma_{i-1,j,k}+\sigma_{i,j+1,k}+\sigma_{i,j-1,k}+\sigma_{i,j,k+1}+\sigma_{i,j,k-1}\right]$$

where $R_{i,j,k}$ contains contributions from pulse, local residual quadrupolar coupling and relaxation mechanisms. Equation (6) is a generalised Bloch equation, modified to describe spin-exchange between neighbouring elementary sub-cells [68]. The set of differential equations described by Equation (6) was solved numerically by using an iterative method [41]. As displayed in Figure 12b, the agreement with experimental data is quantitative, validating our interpretation of this two-time correlation function.

### 3.4. Macroscopic Water Diffusion

**Figure 13:**
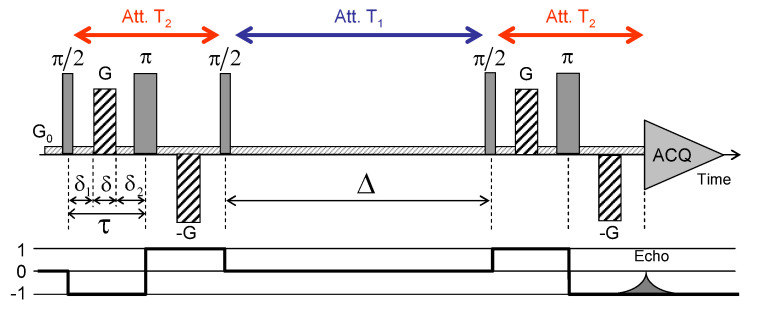
Schematic view of the pulse sequence used to perform pulsed gradient spin-echo (PGSE-NMR) attenuation measurements. Details on the time delays, pulse durations and strengths of the magnetic field gradients *G* are given in the text. Reprinted with permission from [29]. Copyright (2018) American Chemical Society.

Kaolinite compacted sample (dry sample porosity ϕ = 0.52) was selected to investigate water mobility within unswelling aggregates. After saturation of the clay aggregate by pure water, ${}^{1}$H NMR PGSE attenuation measurements were performed to measure the water diffusion tensor inside the sediment (see Section 2). Figure 13 illustrates the pulse sequence used to perform the PGSE NMR attenuation measurements [29]. The encoding of transverse magnetisation is performed by a pulse sequence composed of an initial π/2 pulse used to create transverse magnetisation, a set of bipolar field gradients (duration δ, strength *G*) applied along any desired direction and a final π/2 pulse to transfer back magnetisation in the direction parallel to the static magnetic field $B_{0}$ (called longitudinal direction). After the evolution period (Δ), magnetisation is refocused by using a pulse sequence similar to the encoding one, but with opposite field gradients. That sequence allows probing water mobility along any direction.

Because of the presence of iron (see Section 2), the water relaxation times in saturated sediment ($T_{1}=5.3\times 10^{-2}$ s and $T_{2}=1.2\times 10^{-3}$ s) are much shorter than their value in bulk water ($T_{1}=T_{2}=3$ s). As a consequence, optimisation of the pulse sequence requires limiting the mixing time Δ to 150 ms, i.e., no more than three $T_{1}$ values (see Figure 13). Another limitation of this PGSE pulse sequence originates from the time required to build the magnetic field gradients (typically 60 microseconds). As a consequence, the total duration (4τ = 3.04 ms) of the coding and refocusing pulse sequence (see Figure 13) reaches milliseconds, i.e., the order of magnitude of the transverse relaxation time $T_{2}$ (see above), leading again to important attenuation of the detectable NMR signal. Finally, the intensity of the NMR signal varies according to [75,76]

(7) $$E\left(\vec{G},\Delta \right)=C\;\exp\left[-4\;\pi^{2}\;q^{2}\;{\vec{e}}_{G}^{T}\;D\;{\vec{e}}_{G}\left(\Delta +\frac{3}{2}\tau -\frac{\delta }{6}\right)-\frac{4\tau }{T_{2}}-\frac{\Delta }{T_{1}}\right]$$

where q=γδG/π; γ is the ^1^H gyromagnetic ratio ($\gamma =2.6752\times 10^{8}$ rad/s); G is the strength of the field gradient and ${\vec{e}}_{G}$ its director; D is the water self-diffusion tensor; and δ, Δ and τ are time delays illustrated in Figure 13. Since all measurements are with a unique set of time delays (δ = 500 μs, Δ = 20 ms and τ = 760 μs), the attenuation of the NMR signal satisfies

(8) $$E\left(\vec{G},\Delta \right)=E\left(0,\Delta \right)\;\exp\left[-4\;\pi^{2}\;q^{2}\;{\vec{e}}_{G}^{T}\;D\;{\vec{e}}_{G}\left(\Delta +\frac{3}{2}\tau -\frac{\delta }{6}\right)\right]$$

reducing the signal attenuation to the contribution from the self-diffusion propagator, i.e., the intermediate scattering function probed by QENS [77]. The maximum resolution of these PGSE NMR measurements is limited by the maximum strength of the magnetic field gradient ($G_{max}$ = 1.6 Tesla/m) [76]:

(9) $${\mathrm{resolution}}_{max}=\frac{1}{q_{max}}=\frac{\pi }{\gamma \delta G_{max}}\approx 15\;\mu m$$

**Figure 14:**
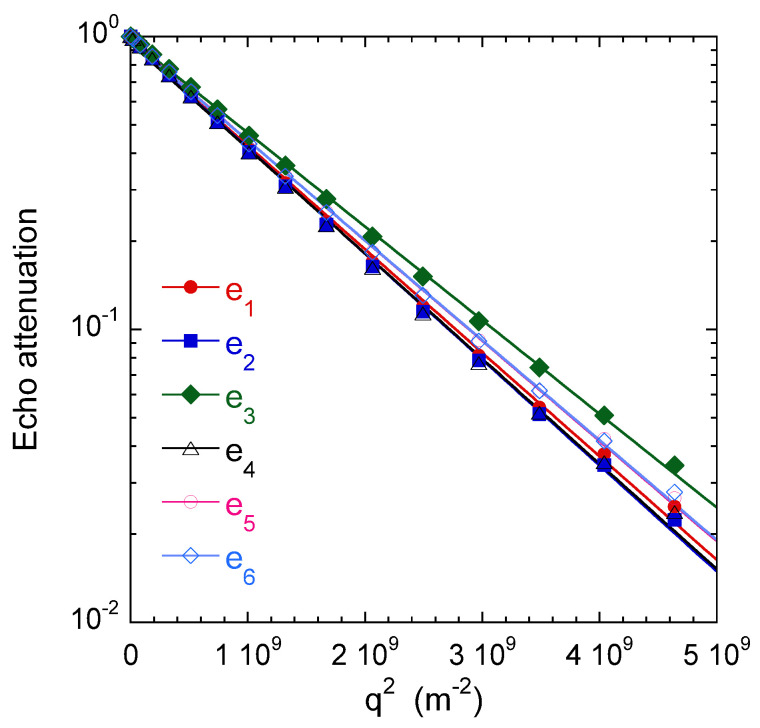
Water self-diffusion propagators measured within the saturated clay sample. More details on the selected diffusion directors $\vec{e_{i}}$ are given (see text above), leading to the components of the self- diffusion tensor $D_{1}=1.041\times 10^{-9}$ m${}^{2}$/s, $D_{2}=1.062\times 10^{-9}$ m${}^{2}$/s, $D_{3}=0.918\times 10^{-9}$ m${}^{2}$/s, $D_{4}=1.067\times 10^{-9}$ m${}^{2}$/s, $D_{5}=0.991\times 10^{-9}$ m${}^{2}$/s, and $D_{6}=0.992\times 10^{-9}$ m${}^{2}$/s. Reprinted with permission from [29]. Copyright (2018) American Chemical Society.

Figure 14 illustrates the results obtained by ^1^H PGSE NMR attenuation measurements within water saturated kaolinite aggregates. The size of the individual kaolinite platelets (∼100 nm) was determined by TEM [29]. It was two orders of magnitude smaller than the maximum resolution of these PGSE measurements. We did not detect significant variation of the self-diffusion coefficient as a function of diffusion time Δ [29].

As displayed in Figure 14, the attenuation perfectly fit a Gaussien relationship, whatever the direction probed by the applied field gradient. Six non-collinear directions of the field gradient were selected and are noted $\vec{e_{1}}=\left(1,0,0\right)$, $\vec{e_{2}}=\left(0,1,0\right)$, $\vec{e_{3}}=\left(0,0,1\right)$, $\vec{e_{4}}=\left(1/\sqrt{2},1/\sqrt{2},0\right)$, $\vec{e_{5}}=\left(1/\sqrt{2},0,1/\sqrt{2}\right)$ and $\vec{e_{6}}=\left(0,1/\sqrt{2},1/\sqrt{2}\right)$, with $\vec{e_{3}}$ oriented parallel to the cylindrical axis of the clay sample.

Said procedure allows determining the principal axes of the self-diffusion tensor and the corresponding eigenvalues, $D_{long}=\left(0.92\pm 0.02\right)\times 10^{-9}$ m${}^{2}$/s and $D_{trans}=\left(1.05\pm 0.02\right)\times 10^{-9}$ m${}^{2}$/s, exhibiting reduced anisotropy of water mobility within the clay sample. Since the longitudinal direction coincides with the compression axis of the clay sample, we can deduce that the small decrease of the water mobility in that direction is induced by the sample preparation. From the water mobility of bulk water $D_{bulk}=\left(2.10\pm 0.02\right)\times 10^{-9}$ m${}^{2}$/s, we can evaluate the tortuosity factor $\theta_{i}$ of the sample resulting from the geometry and tortuosity of its porous network by evaluating the ratio $\theta_{i}=D_{bulk}/D_{i}$, leading to $\theta_{long}=2.28\pm 0.05$ and $\theta_{trans}=2.00\pm 0.05$.

**Figure 15:**
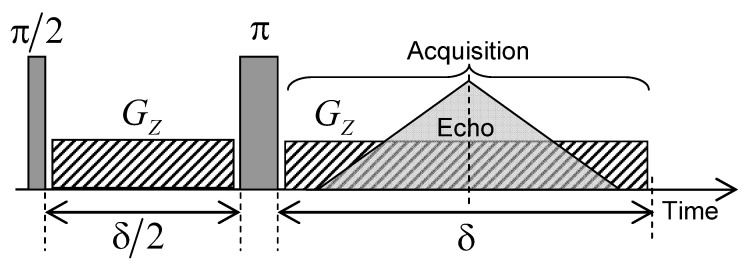
Schematic view of the pulse sequence used to create the water concentration profiles inside the compacted clay sample by magnetic resonance imaging (MRI) technique. Reprinted with permission from [29]. Copyright (2018) American Chemical Society.

**Figure 16:**
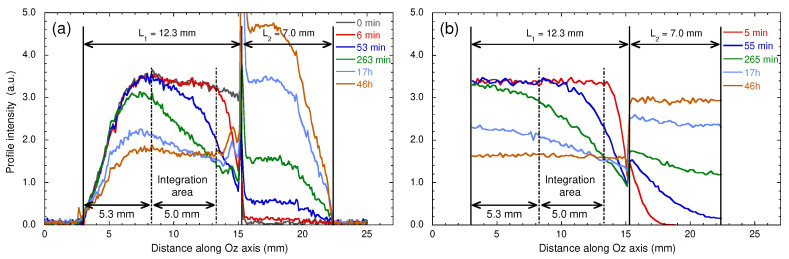
Time evolution of the water concentration profiles: (**a**) measured by MRI after the addition of bulk heavy water on the top of the water saturated sample and (**b**) obtained by numerical simulations of Brownian dynamics (see the text). Reprinted with permission from [29]. Copyright (2018) American Chemical Society.

Nuclear magnetic imaging may be also used to investigate water mobility within the kaolinite porous network in a very long time-scale (up to a few days), better corresponding to macroscopic scales implied in industrial or geological processes. For that purpose, a limited amount of heavy water (0.23 mL) is added on the top of the water saturated kaolinite compacted sample and MRI is used to probe the macroscopic exchange between heavy and natural water by analysing the time evolution of H${}_{2}$O concentration profiles. The MRI measurements were performed on the same Bruker DSX100 spectrometer (see Section 2). The pulse sequence used to perform magnetic resonance imaging (MRI) experiments is illustrated in Figure 15. After an initial π/2 excitation pulse, a magnetic field gradient ($G_{z}$) is applied during a period δ/2 in the vertical direction, parallel to the static magnetic field, thereby corresponding to the cylindrical axis of the sample holder. Inversion pulse (noted π) is then applied and the same field gradient ($G_{z}$) is applied during the period δ with a simultaneous acquisition of the NMR signal. This pulse sequence generates an echo after the evolution period δ/2 leading to a noticeable attenuation ($\exp\left[-\delta /(2T_{2})\right]\approx 0.34$) of the NMR signal pertaining to the confined water molecules without altering the NMR signal from bulk water. These MRI water concentration profiles are recorded with 256 elementary time steps of 10 μs during the evolution period δ, leading to water concentration profiles composed of 256 water layers along the $O_{z}$ direction. In order to simultaneously detect the water molecules confined inside the clay aggregate (length $L_{1}$ = 12.3 mm) and free above it (length $L_{2}$ = 7 mm), we probe a total spatial window ($L_{T}$) of 25 mm, see Figure 16a,b, leading to a spatial resolution ($L_{T}$ / 256) of 97.7 μm. For that purpose, a magnetic field gradient ($G_{z}$) of 0.094 Tesla/m is applied, corresponding to a wave vector $q=1.024\times 10^{4}$ m${}^{-1}$ (see above) leading to the required spatial resolution. In order to improve the signal/noise ratio, the pulse sequence displayed in Figure 15 is repeated 16 times with a time delay of 0.5 s. That repetition delay is much longer than the longitudinal relaxation time of confined water molecules ($T_{1}=5.3\times 10^{-2}$ s), thus optimising acquisition of their NMR signal. By contrast, the NMR signal of bulk water ($T_{1}=3$ s) is then saturated, leading to non-quantitative detection of the bulk water concentration profiles displayed on the right side of Figure 16a.

**Figure 17:**
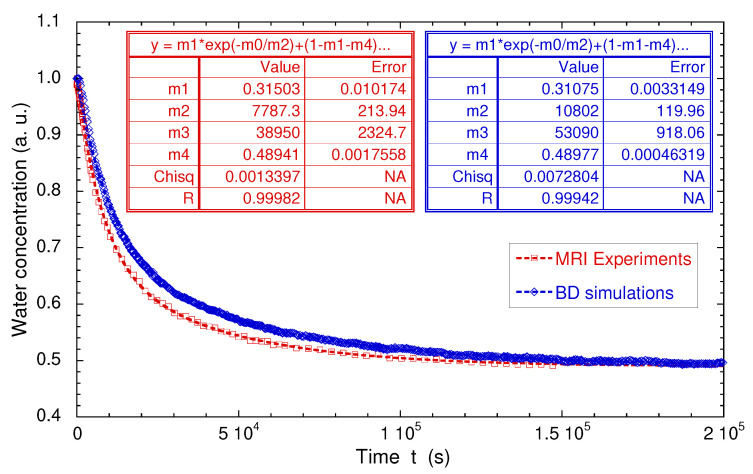
Direct comparison of the time evolutions of the measured and simulated numbers of confined water molecules. Experimental and numerical data are fitted by a biexponential law: $f\left(t\right)=m_{1}\exp\left(-t/m_{2}\right)+\left(1-m_{1}-m_{4}\right)\exp\left(-t/m_{3}\right)+m_{4}$, where $m_{i}$ are constants. Reprinted with permission from [29]. Copyright (2018) American Chemical Society.

Figure 16a displays the water concentration profiles detected after addition of 0.23 mL of bulk water on the top of the kaolinite sample, where the compression was initially applied (see Section 2). As displayed in Figure 16a, significant signal attenuations occur at the lower and upper limits of the spectral window because it exceeds the spatial domain corresponding to complete excitation and detection of the NMR coil. For that purpose, we restrict our analysis by integrating the water concentration profiles over a limited area of 5 mm centred on the clay sample (see Figure 16a). Nevertheless, Figure 16a still exhibits a linear decrease of the initial water concentration profile probably due to compaction implied in the sample preparation (see Section 2), leading to lower porosity at the top of the clay sample, where the compression was applied (see above). Figure 17 exhibits the long-time variation of the partially-integrated water concentration profiles, after normalisation by the initial value. The limiting asymptotic value may be used to evaluate the porosity (noted φ) of the hydrated sample since

(10) $$\mathrm{asymptotic}\;\mathrm{value}=\frac{\mathrm{total}\;H_{2}O}{\mathrm{total}\;H_{2}O+D_{2}O}=\frac{L_{1}\varphi }{L_{2}+L_{1}\varphi }\approx 0.49\pm 0.01$$

where $L_{1}$ (∼12.3 mm) and $L_{2}$ (∼7 mm) are the lengths occupied by the confined hydrated sample and the added bulk water respectively (see above). The corresponding porosity φ=0.55±0.01 is fully compatible with the value determined by picnometry for the compacted sample of dry kaolinite (φ=0.52), since no mechanical constraint is applied during hydration of the kaolinite sample. These measurements are fully validated by numerical simulations of Brownian dynamics—modelling the macroscopic exchange between bulk water and added heavy water (Figure 16b). For that purpose, the water self-diffusion coefficient of the confined water molecules was set equal to the value measured by PGSE in the longitudinal direction; i.e., $0.92\times 10^{-9}$ m${}^{2}$/s (see above). During these BD simulations, the water exchange at the boundary between free and confined water was constricted to insure conservation of the water local density, thereby describing, at a microscopic scale, the incompressibility of liquid water. As displayed in Figure 16a,b and Figure 17, semi-quantitative agreements are obtained between experimental and numerical data, validating the use of MRI concentration profiles to probe such long-time interfacial water exchanges between macroscopic samples of confined and bulk water.

## 4. Conclusions

Multi-scale analysis of the structural and dynamical properties of water molecules confined by clay porous network was determined by a set of complementary nuclear magnetic resonance experiments exploiting spectroscopy, multi-quanta relaxometry, two-time correlation functions, pulsed gradient spin-echo attenuation and magnetic resonance imaging. This whole set of experimental investigations allows one to cover a broad range of characteristic times, varying between microseconds and days. In that context, a complementary set of numerical models, including molecular dynamics, Brownian dynamics and resolution of macroscopic differential equations, was used to validate the interpretation of these multi-scale NMR measurements performed at such various time-scales. For that purpose, bottom-up approaches are applied to fix the values of parameters required by coarse grain modelling from numerical results obtained by simulating more detailed systems. While this study was performed for various clay/water interfaces, including dilute aqueous dispersions and dense sediments or aggregates, it can be extended to other deuterated molecules or quadrupolar ions (^7^Li, ^23^Na, ^133^Cs) confined by various interfacial systems, including zeolites and porous silicates, micelles and membranes, polyelectrolytes and macromolecules.

## Figures and Tables

**Table 1:** Set of characteristic angular velocities ($\lambda_{1}$, $\lambda_{2}$, $\lambda_{3}$) detected by multi-quanta spin-locking relaxometry for ^2^H NMR experiments obtained by varying the irradiation power $\omega_{1}$ and the angle $\theta^{LF}$ (see text).

$\theta^{\mathrm{LF}}$		$0^{{}^{\circ}}$	$30^{{}^{\circ}}$	$90^{{}^{\circ}}$		$0^{{}^{\circ}}$	$30^{{}^{\circ}}$	$90^{{}^{\circ}}$		$0^{{}^{\circ}}$	$30^{{}^{\circ}}$	$90^{{}^{\circ}}$
$\omega_{1}$**(10**${}^{5}$ **rad/s)**		$\lambda_{1}$**(10**${}^{5}$ **rad/s)**				$\lambda_{2}$**(10**${}^{5}$ **rad/s)**				$\lambda_{3}$**(10**${}^{5}$ **rad/s)**		
1.122		2.46	2.28	2.22		1.60	1.36	1.23		0.86	0.86	0.86
0.561		1.48	1.29	1.36		1.11	0.86	0.86		0.37	0.37	0.43
0.280		1.05	0.80	0.80		0.92	0.68	0.62		0.18	0.18	0.09
0.140		0.86	0.55	0.55		0.80	0.62	0.49		0.06	0.06	0.09
0.070		0.80	0.55	0.43		0.80	0.55	0.43		0.06	0.06	0.03

**Figure A1:**
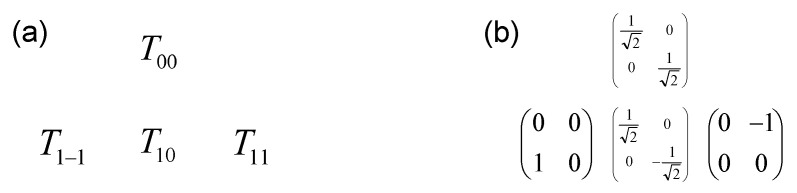
Complete orthogonal basis set (four elements) describing the evolution of the spin I = 1/2 (Pauli matrices): (**a**) symbolic representation $T_{ij}$; (**b**) explicit matrix representation of irreducible tensor operators.

**Figure A2:**
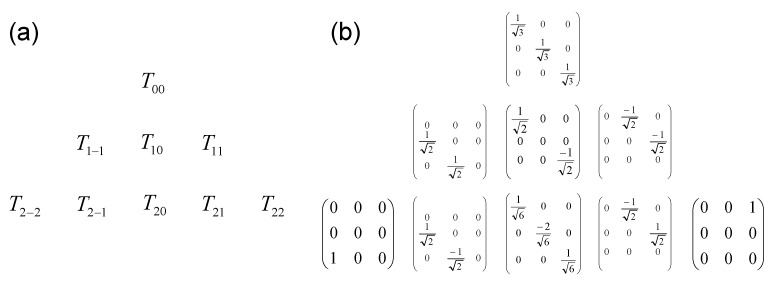
Complete orthogonal basis set (nine elements) describing the evolution of the spin I = 1: (**a**) symbolic representation $T_{ij}$; (**b**) explicit matrix representation of irreducible tensor operators.

## Abbreviations

The following abbreviations are used in this manuscript:

EFG	Electric Field Gradient
GCMC	Grand Canonical Monte Carlo
INS	Inelastic Neutron scattering
MRI	Magnetic Resonance Imaging
PGSE	Pulsed-Gradient Spin-Echo
QENS	Quasi Elastic Neutron Scattering
TEM	Transmission Electron Microscopy

## Appendix A. Quadrupolar and Heteronuclear Dipolar Hamiltonian

The quadrupolar Hamiltonian [33,78,79] is defined by:

(A1) $$H_{Q}=C_{Q}\;\sum_{p=-2}^{2}\;{\left(-1\right)}^{p}\;F_{2,-p}^{Q,L}\;T_{2,p}^{IR}\;\mathrm{with}\;C_{Q}=\sqrt{\frac{3}{2}}\;\frac{e\;Q\;(1+\gamma_{\infty })}{I(2I-1)\;\hbar }$$

where *e* is the electron charge, *Q* is the quadrupolar moment of the nuclei [80] and $\left(1+\gamma_{\infty }\right)$ is the Steinhermer antishielding factor [80]. In the above equation,

(A2a) $$\begin{array}{c} F_{2,0}^{Q,L}=\frac{1}{2}\;V_{zz}^{L}\;,\;F_{2,\pm 1}^{Q,L}=\mp \frac{1}{\sqrt{6}}\;\left(V_{xz}^{L}\pm iV_{yz}^{L}\right)\;,\;F_{2,\pm 2}^{Q,L}=\frac{1}{2\;\sqrt{6}}\;\left(V_{xx}^{L}-V_{yy}^{L}\pm 2iV_{xy}^{L}\right) \end{array}$$

and

(A2b) $$\begin{array}{c} T_{2,0}^{Q,IR}=\frac{1}{\sqrt{6}}\;\left(3I_{z}^{2}-I\left(I+1\right)\right)\;,\;T_{2,\pm 1}^{Q,IR}=\mp \frac{1}{2}\;\left(I_{z}I_{\pm }+I_{\pm }I_{z}\right)\;,\;T_{2,\pm 2}^{Q,IR}=\frac{1}{2}\;I_{\pm }^{2} \end{array}$$

where $V_{\alpha \beta }^{L}$ are the components of the electric field gradient (EFG) evaluated in the laboratory frame (noted *L*); $T_{2,\pm p}^{Q,IR}$ (for *p* = −2 to 2) are the second order irreducible tensors operators; $I_{x}$, $I_{y}$ and $I_{z}$ are the spin operators; and $I_{\pm }=I_{x}\pm iI_{y}$.

In the presence of a static quadrupolar coupling, the equidistant Zeeman energy levels are modified by the residual quadrupolar coupling, leading to a quadrupolar splitting of the resonance lines according to:

(A3) $$\omega_{m-1,m}=\sqrt{\frac{3}{8}}\;C_{Q}\;\langle V_{zz}^{L}\rangle \left(1-2m\right)$$

for *m* varying between *I* and −I+1.

During a change of frame, the components of the EFG transform like the second order spherical harmonics [33]:

(A4) $$F_{2,q}^{Q,L}=\sum_{p=-2}^{2}\;F_{2,p}^{Q,P}\;D_{p,q}^{LP}\left(\theta ,\varphi ,\psi \right)$$

with $D_{p,q}^{LP}\left(\theta ,\varphi ,\psi \right)$, the components of the Wigner rotation matrices [33] where the set of (θ,φ,ψ) Euler angles defines the orientation, into the static magnetic field, of the principal axis of the tensor describing the EFG felt by the quadrupolar nucleus. Three sets of frames are useful to describe the orientation of the principal component of the EFG: the laboratory frame (noted *L*), a frame attached to the dense clay sediment (noted *F*) and a frame attached to the individual quadrupolar nucleus (noted *P*; see Figure 2c)). The **e**${}_{z}$ directors of these different frames are, respectively, the direction of the static magnetic field $B_{0}$ (laboratory frame *L*), the normal to the clay sediment **n** (sediment frame *F*) and the director of the principal component of the EFG, noted $V_{zz}^{P}$ (particle frame *P*; see Figure 2c)).

The measured quadrupolar splitting is derived from Equations (A3) and (A4):

(A5) $$\begin{array}{cc} \omega_{m-1,m}^{app}\; & =\;A_{m}\;V_{zz}^{P}\;\sum_{p=-2}^{2}\;D_{p,0}^{LF}\left(\theta^{LF},\varphi^{LF},\psi^{LF}\right)\;\langle D_{0,p}^{FP}\left(\theta^{FP},\varphi^{FP},\psi^{FP}\right)\;\rangle \\ \; & \;\mathrm{with}\;A_{m}=\frac{3eQ\left(1+\gamma_{\infty }\right)\;\left(1-2m\right)}{4I\;(2I-1)\;\hbar } \end{array}$$

The angular average is evaluated in Equation (A5) over all the orientations of EFG principal component within the sediment. The first set of Wigner rotation matrix describes the orientation of the macroscopic clay film with respect to the magnetic field $B_{0}$ and the Wigner rotation matrix in the bracket characterizes the average orientation of EFG principal component within the sediment (see Figure 2c). For clay sediments with cylindrical symmetry, only the component p=0 contributes to Equation (A5) which reduces to:

(A6) $$\omega_{m-1,m}^{app}\;=\;A_{m}\;V_{zz}^{P}\;\frac{3\cos^{2}\theta^{LF}-1}{2}\;\langle \frac{3\cos^{2}\theta^{FP}-1}{2}\rangle$$

For deuterium atoms pertaining to heavy water, the electrostatic field gradient $V_{zz}^{P}$ is oriented along the $\vec{OD}$ director of the water molecule and corresponds to quadrupolar coupling constant $\omega_{Q}=990\times 10^{3}$ rad/s [59].

In addition to the quadrupolar coupling, the heteronuclear dipolar coupling may also be responsible for the NMR relaxation of the confined probes because of the presence of paramagnetic impurities. The corresponding heteronuclear dipolar Hamiltonian [33,78,79] becomes:

(A7) $$H_{D}\left(t\right)=C_{D}\;\sum_{m=-2}^{2}\;{\left(-1\right)}^{m}\;T_{2,m}^{D,IR}\;F_{2,-m}^{D,L}\left(t\right)\;\;\mathrm{with}\;C_{D}=-\frac{\mu_{0}}{4\pi }\;\gamma_{I}\;\gamma_{S}\;\hbar$$

where the $C_{D}$ is the dipolar coupling constant and the spin operators become [33,78,79]:

(A8) $$T_{2,0}^{D,IR}=\frac{1}{\sqrt{6}}\left(2I_{z}S_{z}-\frac{1}{2}\left(I_{+}S_{-}+I_{-}S_{+}\right)\right),\;T_{2,\pm 1}^{D,IR}=\mp \frac{1}{2}\left(I_{z}S_{\pm }+I_{\pm }S_{z}\right)\;\mathrm{and}\;T_{2,\pm 2}^{D,IR}=\frac{1}{2}\;I_{\pm }S_{\mp }$$

The functions $F_{2,m}^{D,L}\left(t\right)$ in Equation (A7) are related to the second order spherical harmonics describing the reorientation of the vector joining the two coupled spins (noted ${\vec{r}}_{IS}\left(t\right)$) by reference with the static magnetic field [33,78,79]:

(A9) $$F_{2,-m}^{D,L}\left(t\right)\;=\;\sqrt{\frac{24\pi }{5}}\;\frac{Y_{2,-m}\left(\theta ,\varphi \right)}{r_{IS}^{3}}$$

## Appendix B. NMR Relaxation Theory

In the framework of the Redfield theory [81], the time evolution of the spin quantum states, also called coherences, is described by the master equation [33,78,79]:

(A10) $$\frac{d\sigma^{*}}{dt}=-i\;\left[H_{S}^{*},\sigma^{*}\right]+f\left(\sigma^{*}\right)$$

As noted by the asterisk (${}^{*}$), all terms are evaluated in the Larmor frequency rotating frame. The commutator describes the contribution from the static Hamiltonians $H_{S}^{*}$, including the excitation pulses and the residual quadrupolar Hamiltonian. The second term describes the contribution from the fluctuating parts of the quadrupolar and dipolar Hamiltonians:

(A11) $$H_{XF}^{*}\left(t\right)=C_{X}\;\sum_{m=-2}^{2}\;{\left(-1\right)}^{m}\;T_{2,m}^{X,IR}\;e^{im\omega_{0}t}\;\left(F_{2,-m}^{X,L}\left(t\right)\;-\;\langle F_{2,-m}^{X,L}\left(t\right)\rangle \right)$$

where the index *X* stands for the various relaxation mechanisms (i.e., *Q* or *D*). This last contribution to the master equation is given by [33,78,79,81]:

(A12) $$f\left(\sigma^{*}\right)=\;\int_{0}^{t_{sup}}\langle \;\left[H_{XF}^{*}\left(t\right),\left[e^{-iH_{S}^{*}\tau }\;H_{XF}^{*+}\left(t-\tau \right)\;e^{iH_{S}^{*}\tau }\;,\;\sigma^{*}\left(t\right)\right]\;\right]\rangle \;d\tau$$

If the time-scales characterising the decorrelation of the various Hamiltonians are much smaller than the time-scale sampled by the evolution of the coherences, the upper limit of the integral $t_{sup}$ in Equation (A12) may be set equal to infinity. This hypothesis restricts the validity of the Redfield theory applied to NMR relaxation [33,78,79].

Let us introduce the autocorrelation functions of the fluctuating components of the Hamiltonian

(A13) $$\begin{array}{cc} G_{m}^{X,L}\left(\tau \right)= & \langle \left(F_{2,m}^{X,L}\left(0\right)-<F_{2,m}^{X,L}>\right)\times \left(F_{2,m}^{X,L}\left(\tau \right)-<F_{2,m}^{X,L}>\right)\rangle \\ \; & +\langle \left(F_{2,-m}^{X,L}\left(0\right)-<F_{2,-m}^{X,L}>\right)\times \left(F_{2,-m}^{X,L}\left(\tau \right)-<F_{2,-m}^{X,L}>\right)\rangle \;\mathrm{with}\;m\in \left\{0,1,2\right\} \end{array}$$

By neglecting the time evolution of the coherences during the irradiation pulses, Equation (A12) becomes then [33,78,79]:

(A14) $$f\left(\sigma^{*}\right)=\;-\sum_{m=0}^{2}\;\left[T_{2,m}^{X,IR},\left[\;T_{2,-m}^{X,IR},\sigma^{*}\right]\right]J_{m}^{X,L}\left(m\omega_{0}\right)$$

where the so-called spectral densities $J_{m}^{X,L}\left(m\omega_{0}\right)$ satisfy the relationship [33,78,79]:

(A15) $$J_{m}^{X,L}\left(m\omega_{0}\right)=-C_{X}^{2}\;\int_{0}^{\infty }\;G_{m}^{X,L}\left(t\right)\;e^{-im\omega_{0}t}\;dt$$

The above-mentioned approximation is generally valid for classical relaxation measurements because the duration of the detection pulses (typically a few μs) is much shorter than the time evolution of the coherences. Finally, a complete basis set of coherences is required to translate Equation (A14) into a matrix form [64,82,83,84,85].

In the case of spin-locking relaxation measurements, one cannot neglect the time evolution of the coherences during the irradiation power, since it is applied during all the evolution period of the coherences. As a consequence, the time evolution of the dipolar and quadrupolar couplings under the influence of the static Hamiltonian $H_{S}^{*}$ must be taken into account in Equation (A12) as described implicitly by the term $e^{-iH_{S}^{*}\tau }\;H_{XF}^{*+}\left(t-\tau \right)\;e^{iH_{S}^{*}\tau }$ in the double commutator (see Equation (A12)). For that purpose, the static Hamiltonian is also formulated in a matrix form by using the complete basis set of coherences [20,42,44]. After evaluating its eigenvalues (noted $\pm i\lambda_{p}$) and corresponding eigenvectors (noted ${\vec{v}}_{p}$), one obtains a new complete basis set. The problem is then easily solved by projecting, into this eigenvectors basis set, the initial basis set of the coherences used to describe the $T_{2,m}^{X,IR}$ spin operators.

By using the Wigner rotation matrices [35] (cf. Equation (A4)), it is possible to relate the derivation of the *apparent* correlation functions $G_{m}^{X,L}\left(\tau \right)$, evaluated in the laboratory frame (noted *L*), with their *intrinsic* value evaluated in the frame attached to the clay sediment (noted *F*) [86]:

(A16a) $$\begin{array}{cc} G_{0}^{X,L}\left(\tau \right) & =\frac{{\left(1-3\cos^{2}\theta^{LF}\right)}^{2}}{4}\;G_{0}^{X,F}\left(\tau \right)+3\cos^{2}\theta^{LF}\sin^{2}\theta^{LF}\;G_{1}^{X,F}\left(\tau \right)+\frac{3{\left(1-3\cos^{2}\theta^{LF}\right)}^{2}}{4}\;G_{2}^{X,F}\left(\tau \right) \end{array}$$

(A16b) $$\begin{array}{cc} G_{1}^{X,L}\left(\tau \right) & =\frac{3\cos^{2}\theta^{LF}\sin^{2}\theta^{LF}}{2}\;G_{0}^{X,F}\left(\tau \right)+\frac{1-3\cos^{2}\theta^{LF}+4\cos^{4}\theta^{LF}}{2}\;G_{1}^{X,F}\left(\tau \right)+\frac{1-\cos^{4}\theta^{LF}}{2}\;G_{2}^{X,F}\left(\tau \right) \end{array}$$

(A16c) $$\begin{array}{cc} G_{2}^{X,L}\left(\tau \right) & =\frac{3{\left(1-\cos^{2}\theta^{LF}\right)}^{2}}{8}\;G_{0}^{X,F}\left(\tau \right)+\frac{1-\cos^{4}\theta^{LF}}{2}\;G_{1}^{X,F}\left(\tau \right)+\frac{1+6\cos^{2}\theta^{LF}+\cos^{4}\theta^{LF}}{8}\;G_{2}^{X,F}\left(\tau \right) \end{array}$$

Obviously, the same relationship may be deduced for the corresponding spectral densities thanks to the linearity of the Fourier transform (see Equation (A15)).

## Appendix C. Matrix Representation of the Irreducible Tensor Operators

Depending on the spin state, complete orthonormal basis sets may be constructed by using the irreducible tensor operators, also called coherences [87,88,89]. Symmetric and antisymmetric combinations of the coherences [83,84,85] are also introduced:

(A17) $$T_{lp}\left(s\right)=\frac{1}{\sqrt{2}}\left(T_{l-p}+T_{lp}\right)\;\mathrm{and}\;T_{lp}\left(a\right)=\frac{1}{\sqrt{2}}\left(T_{l-p}-T_{lp}\right)$$

By using these new coherences, the three spin operators, $I_{x}$, $I_{y}$ and $I_{z}$, become proportional to $T_{11}\left(a\right)$, $T_{11}\left(s\right)$ and $T_{10}$ respectively, simplifying the formulation of the Hamiltonians describing the irradiation pulse and the heteronuclear dipolar coupling (see Equation (A8)).

As displayed in Figure A1 and Figure A2, the size of the basis set increases significantly as a function of the spin of the nucleus.

## Appendix D. Application to the Relaxation of Quadrupolar Nuclei

The differential equation describing the time evolution of the coherences (Equations (A10)–(A15)) may be written in a matrix form. To simplify the derivations of these matrices, we selected to use symmetric and antisymmetric combinations of the coherences [83,84,85]; see Equation (A17). By using these coherences, the three spin operators, $I_{x}$, $I_{y}$ and $I_{z}$, become proportional to $T_{11}\left(a\right)$, $T_{11}\left(s\right)$ and $T_{10}$ respectively, simplifying the formulation of the Hamiltonians describing the irradiation pulse and the heteronuclear dipolar coupling (see Equations (A7)–(A9)).

For spin I = 1 nuclei, the time evolution of the coherences under the influence of the static Hamiltonian (noted $H_{S}^{*}$ in Equation (A10)) including the residual quadrupolar coupling ($\omega_{Q}$) and the irradiation pulse ($\omega_{1}$) becomes [64,83]:

(A18) $$\frac{d}{dt}\left(\begin{array}{c} T_{20}\\ T_{11}\left(a\right)\\ T_{21}\left(s\right)\\ T_{22}\left(s\right)\\ T_{10}\\ T_{11}\left(s\right)\\ T_{21}\left(a\right)\\ T_{22}\left(a\right) \end{array}\right)=i\left(\begin{array}{cccccccc} 0 & 0 & -\sqrt{3}\;\omega_{1} & 0 & 0 & 0 & 0 & 0\\ 0 & 0 & \omega_{Q} & 0 & 0 & 0 & 0 & 0\\ -\sqrt{3}\;\omega_{1} & \omega_{Q} & 0 & -\omega_{1} & 0 & 0 & 0 & 0\\ 0 & 0 & -\omega_{1} & 0 & 0 & 0 & 0 & 0\\ 0 & 0 & 0 & 0 & 0 & -\omega_{1} & 0 & 0\\ 0 & 0 & 0 & 0 & -\omega_{1} & 0 & -\omega_{Q} & 0\\ 0 & 0 & 0 & 0 & 0 & -\omega_{Q} & 0 & -\omega_{1}\\ 0 & 0 & 0 & 0 & 0 & 0 & -\omega_{1} & 0 \end{array}\right)\left(\begin{array}{c} T_{20}\\ T_{11}\left(a\right)\\ T_{21}\left(s\right)\\ T_{22}\left(s\right)\\ T_{10}\\ T_{11}\left(s\right)\\ T_{21}\left(a\right)\\ T_{22}\left(a\right) \end{array}\right)$$

leading to two independent sub-sets of coherences. Analytical derivation of the eigenvalues $\pm i\lambda_{p}$ with p∈{0,⋯,3} and their corresponding eigenvectors ${\vec{v}}_{p}$ are used to derive the general solutions of Equation (A18) [20,83]. Among others, two coherences are of practical interest for the interpretation of spin-locking relaxation measurements:

(A19a) $$\begin{array}{cc} \begin{array}{cc} e^{iH_{S}^{*}\tau }T_{20}e^{-iH_{S}^{*}\tau }= & \sqrt{3}\omega_{1}\omega_{Q}\frac{1-\cos(\lambda_{1}\tau )}{\lambda_{1}^{2}}T_{11}\left(a\right)+\frac{\omega_{Q}^{2}+\omega_{1}^{2}\left(1-3\cos\left(\lambda_{1}\tau \right)\right)}{\lambda_{1}^{2}}T_{20}\\ \; & -i\sqrt{3}\omega_{1}\frac{\sin(\lambda_{1}\tau )}{\lambda_{1}}T_{21}\left(s\right)+\sqrt{3}\omega_{1}^{2}\frac{\cos(\lambda_{1}\tau )-1}{\lambda_{1}^{2}}T_{22}\left(s\right) \end{array}\\ \mathrm{and}\; & \end{array}$$

(A19b) $$\begin{array}{c} \begin{array}{cc} e^{iH_{S}^{*}\tau }T_{10}e^{-iH_{S}^{*}\tau }= & \frac{\lambda_{2}\cos\left(\lambda_{3}\tau \right)+\lambda_{3}\cos\left(\lambda_{2}\tau \right)}{\lambda_{1}}T_{10}-i\omega_{1}\frac{\sin\left(\lambda_{3}\tau \right)+\sin\left(\lambda_{2}\tau \right)}{\lambda_{1}}T_{11}\left(s\right)\\ \; & +\omega_{1}\frac{\cos\left(\lambda_{3}\tau \right)-\cos\left(\lambda_{2}\tau \right)}{\lambda_{1}}T_{21}\left(a\right)-i\frac{\lambda_{2}\sin\left(\lambda_{3}\tau \right)-\lambda_{3}\sin\left(\lambda_{2}\tau \right)}{\lambda_{1}}T_{22}\left(a\right) \end{array} \end{array}$$

where the characteristic angular velocities $\lambda_{0}$, $\lambda_{1}$, $\lambda_{2}$ and $\lambda_{3}$ are defined by:

(A20) $$\lambda_{0}=0,\;\lambda_{1}=\sqrt{\omega_{Q}^{2}+4\omega_{1}^{2}},\;\lambda_{2}=\frac{\lambda_{1}+\omega_{Q}}{2},\;\mathrm{and}\;\lambda_{3}=\frac{\lambda_{1}-\omega_{Q}}{2}\;\mathrm{respectively}.$$

As a consequence, under the simultaneous influences of the residual quadrupolar coupling ($\omega_{Q}$) and the irradiation pulse ($\omega_{1}$), the $T_{20}$ coherence oscillates according to the angular velocity $\lambda_{1}$ while two angular velocities ($\lambda_{2}$ and $\lambda_{3}$) drive the oscillations of the $T_{10}$ coherence.

Straightforward calculations of the set of Equations (A14) and (A15) lead to the contributions of the quadrupolar [83] and heteronuclear dipolar [64] relaxation mechanisms to the time evolution of the coherences.

(A21) $$\frac{d}{dt}\left(\begin{array}{c} T_{20}\\ T_{11}\left(a\right)\\ T_{21}\left(s\right)\\ T_{22}\left(s\right)\\ T_{10}\\ T_{11}\left(s\right)\\ T_{21}\left(a\right)\\ T_{22}\left(a\right) \end{array}\right)=-\mathrm{diag}\;\left(A,B,C,D,E,B,C,D\right)\times \left(\begin{array}{c} T_{20}\\ T_{11}\left(a\right)\\ T_{21}\left(s\right)\\ T_{22}\left(s\right)\\ T_{10}\\ T_{11}\left(s\right)\\ T_{21}\left(a\right)\\ T_{22}\left(a\right) \end{array}\right)$$

where $A=A^{Q}+A^{D}$, $B=B^{Q}+B^{D}$, $C=C^{Q}+C^{D}$, $D=D^{Q}+D^{D}$ and $E=E^{Q}+E^{D}$, with

(A22a) $$\begin{array}{c} \begin{array}{cc} A^{Q} & =3J_{1}^{Q}\left(\omega_{1}\right)\;\mathrm{and}\\ A^{D} & =\frac{J_{0}^{D}\left(\omega_{0}-\omega_{S}\right)}{3}+J_{1}^{D}\left(\omega_{0}\right)+2J_{2}^{D}\left(\omega_{0}+\omega_{S}\right) \end{array} \end{array}$$

(A22b) $$\begin{array}{c} \begin{array}{cc} B^{Q} & =\frac{3J_{0}^{Q}\left(0\right)}{2}+\frac{5J_{1}^{Q}\left(\omega_{0}\right)}{2}+J_{2}^{Q}\left(2\omega_{0}\right)\;\mathrm{and}\\ B^{D} & =\frac{2J_{0}^{D}\left(0\right)}{9}+\frac{J_{0}^{D}\left(\omega_{0}-\omega_{S}\right)}{18}+\frac{J_{1}^{D}\left(\omega_{0}\right)}{6}+\frac{J_{1}^{D}\left(\omega_{S}\right)}{3}+\frac{J_{2}^{D}\left(\omega_{0}+\omega_{S}\right)}{3} \end{array} \end{array}$$

(A22c) $$\begin{array}{c} \begin{array}{cc} C^{Q} & =\frac{3J_{0}^{Q}\left(0\right)}{2}+\frac{J_{1}^{Q}\left(\omega_{0}\right)}{2}+J_{2}^{Q}\left(2\omega_{0}\right)\;\mathrm{and}\\ C^{D} & =\frac{2J_{0}^{D}\left(0\right)}{9}+\frac{5J_{0}^{D}\left(\omega_{0}-\omega_{S}\right)}{18}+\frac{5J_{1}^{D}\left(\omega_{0}\right)}{6}+\frac{J_{1}^{D}\left(\omega_{S}\right)}{3}+\frac{5J_{2}^{D}\left(\omega_{0}+\omega_{S}\right)}{3} \end{array} \end{array}$$

(A22d) $$\begin{array}{c} \begin{array}{cc} D^{Q} & =J_{1}^{Q}\left(\omega_{0}\right)+2J_{2}^{Q}\left(2\omega_{0}\right)\;\mathrm{and}\\ D^{D} & =\frac{8J_{0}^{D}\left(0\right)}{9}+\frac{J_{0}^{D}\left(\omega_{0}-\omega_{S}\right)}{9}+\frac{J_{1}^{D}\left(\omega_{0}\right)}{3}+\frac{4J_{1}^{D}\left(\omega_{S}\right)}{3}+\frac{2J_{2}^{D}\left(\omega_{0}+\omega_{S}\right)}{3} \end{array} \end{array}$$

(A22e) $$\begin{array}{c} \begin{array}{cc} E^{Q} & =J_{1}^{Q}\left(\omega_{0}\right)+4J_{2}^{Q}\left(2\omega_{0}\right)\;\mathrm{and}\\ E^{D} & =\frac{J_{0}^{D}\left(\omega_{0}-\omega_{S}\right)}{9}+\frac{J_{1}^{D}\left(\omega_{0}\right)}{3}+\frac{2J_{2}^{D}\left(\omega_{0}+\omega_{S}\right)}{3} \end{array} \end{array}$$

Under slow modulation of the quadrupolar and heteronuclear dipolar couplings, i.e., when:

(A23) $$J_{0}^{X}\left(0\right)>>J_{m}^{X}\left(\omega_{0}\right)>>J_{m}^{X}\left(\omega_{S}\right)\;\mathrm{with}\;m\in \left\{1,2\right\}\;\mathrm{and}\;X\in \left\{Q,D\right\}$$

the set of Equations (A22a)–(A22e) reduces to [21,22]:

(A24) $$\begin{array}{cc} A & =3U_{Q}+U_{D}\\ B & =C=\frac{3J_{0}^{Q}\left(0\right)}{2}+\frac{5U_{Q}}{2}+\frac{2J_{0}^{D}\left(0\right)}{9}+\frac{U_{D}}{2}\\ D & =3U_{Q}+\frac{8J_{0}^{D}\left(0\right)}{9}+U_{D}\\ E & =5U_{Q}+\frac{U_{D}}{3}\\ \mathrm{where}\;U_{Q}= & J_{1}^{Q}\left(\omega_{0}\right)\approx J_{2}^{Q}\left(2\omega_{0}\right)\;\mathrm{and}\;U_{D}=\frac{J_{0}^{D}\left(\omega_{S}-\omega_{0}\right)}{3}+J_{1}^{D}\left(\omega_{0}\right)+2J_{1}^{D}\left(\omega_{S}+\omega_{0}\right) \end{array}$$

Four independent measurements of the relaxation of the $T_{20}$, $T_{11}$, $T_{22}$ and $T_{10}$ coherences lead then to the four dominant contributions ($U_{Q}$, $J_{0}^{Q}\left(0\right)$, $U_{D}$ and $J_{0}^{D}\left(0\right)$) quantifying the quadrupolar and heteronuclear dipolar relaxation mechanisms [21,22].

The derivation of the time evolution of the coherences under spin-locking condition requires taking into account the evolution of the fluctuating part of the quadrupolar [83] and dipolar [20] Hamiltonians under the influence of the static Hamiltonians, as described by the term $e^{-iH_{S}^{*}\tau }\;H_{XF}^{*+}\left(t-\tau \right)\;e^{iH_{S}^{*}\tau }$ in Equation (A12). In the next approximation, we focus only on the m=0 component of the fluctuating Hamiltonians because their m=1 and m=2 components oscillate at angular velocities ($\omega_{0}$ and $2\omega_{0}$) (cf. Equation (A14)) much larger than the characteristic angular velocities ($\lambda_{i}$) (cf. Equations (A19a)–(A20)). By using Equations (A19a) and (A19b), that approximation leads to:

(A25) $$\frac{d}{dt}\left(\begin{array}{c} T_{20}\\ T_{11}\left(a\right)\\ T_{21}\left(s\right)\\ T_{22}\left(s\right)\\ T_{10}\\ T_{11}\left(s\right)\\ T_{21}\left(a\right)\\ T_{22}\left(a\right) \end{array}\right)=-\left(\begin{array}{cccccccc} A & -\sqrt{3}K & 0 & 0 & 0 & 0 & 0 & 0\\ 0 & B & 0 & 2K^{D} & 0 & 0 & 0 & 0\\ 0 & 0 & C & 0 & 0 & 0 & 0 & 0\\ 0 & -K^{Q}+K^{D} & 0 & D & 0 & 0 & 0 & 0\\ 0 & 0 & 0 & 0 & E & 0 & -K & 0\\ 0 & 0 & 0 & 0 & 0 & L & 0 & 2K^{D}\\ 0 & 0 & 0 & 0 & 0 & 0 & M & 0\\ 0 & 0 & 0 & 0 & 0 & -K^{Q}+K^{D} & 0 & D \end{array}\right)\left(\begin{array}{c} T_{20}\\ T_{11}\left(a\right)\\ T_{21}\left(s\right)\\ T_{22}\left(s\right)\\ T_{10}\\ T_{11}\left(s\right)\\ T_{21}\left(a\right)\\ T_{22}\left(a\right) \end{array}\right)$$

with

(A26a) $$\begin{array}{c} \begin{array}{cc} A^{Q} & =3J_{1}^{Q}\left(\omega_{0}\right)\;\mathrm{and}\;\\ A^{D} & =\frac{J_{0}^{D}\left(\omega_{0}-\omega_{S}\right)}{3}+J_{1}^{D}\left(\omega_{0}\right)+2J_{2}^{D}\left(\omega_{0}+\omega_{S}\right) \end{array} \end{array}$$

(A26b) $$\begin{array}{c} \begin{array}{cc} B^{Q} & =\frac{3\omega_{Q}^{2}J_{0}^{Q}\left(0\right)+4\omega_{1}^{2}J_{0}^{Q}\left(\lambda_{1}\right)}{2\lambda_{1}^{2}}+\frac{5J_{1}^{Q}\left(\omega_{0}\right)}{2}+J_{2}^{Q}\left(2\omega_{0}\right)\;\mathrm{and}\;\\ B^{D} & =\frac{2\left(\lambda_{2}J_{0}^{D}\left(\lambda_{3}\right)+\lambda_{3}J_{0}^{D}\left(\lambda_{2}\right)\right)}{9\lambda_{1}}+\frac{J_{0}^{D}\left(\omega_{0}-\omega_{S}\right)}{18}+\frac{J_{1}^{D}\left(\omega_{0}\right)}{6}+\frac{J_{1}^{D}\left(\omega_{S}\right)}{3}+\frac{J_{2}^{D}\left(\omega_{0}+\omega_{S}\right)}{3} \end{array} \end{array}$$

(A26c) $$\begin{array}{c} \begin{array}{cc} C^{Q} & =\frac{3\omega_{Q}^{2}J_{0}^{Q}\left(0\right)+4\omega_{1}^{2}J_{0}^{Q}\left(\lambda_{1}\right)}{2\lambda_{1}^{2}}+\frac{J_{1}^{Q}\left(\omega_{0}\right)}{2}+J_{2}^{Q}\left(2\omega_{0}\right)\;\mathrm{and}\;\\ C^{D} & =\frac{2\left(\lambda_{2}J_{0}^{D}\left(\lambda_{3}\right)+\lambda_{3}J_{0}^{D}\left(\lambda_{2}\right)\right)}{9\lambda_{1}}+\frac{5J_{0}^{D}\left(\omega_{0}-\omega_{S}\right)}{18}+\frac{5J_{1}^{D}\left(\omega_{0}\right)}{6}+\frac{J_{1}^{D}\left(\omega_{S}\right)}{3}+\frac{5J_{2}^{D}\left(\omega_{0}+\omega_{S}\right)}{3} \end{array} \end{array}$$

(A26d) $$\begin{array}{c} \begin{array}{cc} D^{Q} & =J_{1}^{Q}\left(\omega_{0}\right)+2J_{2}^{Q}\left(2\omega_{0}\right)\;\mathrm{and}\;\\ D^{D} & =\frac{8J_{0}^{D}\left(0\right)}{9}+\frac{J_{0}^{D}\left(\omega_{0}-\omega_{S}\right)}{9}+\frac{J_{1}^{D}\left(\omega_{0}\right)}{3}+\frac{4J_{1}^{D}\left(\omega_{S}\right)}{3}+\frac{2J_{2}^{D}\left(\omega_{0}+\omega_{S}\right)}{3} \end{array} \end{array}$$

(A26e) $$\begin{array}{c} \begin{array}{cc} E^{Q} & =J_{1}^{Q}\left(\omega_{0}\right)+4J_{2}^{Q}\left(2\omega_{0}\right)\;\mathrm{and}\;\\ E^{D} & =\frac{J_{0}^{D}\left(\omega_{0}-\omega_{S}\right)}{9}+\frac{J_{1}^{D}\left(\omega_{0}\right)}{3}+\frac{2J_{2}^{D}\left(\omega_{0}+\omega_{S}\right)}{3} \end{array} \end{array}$$

(A26f) $$\begin{array}{c} \begin{array}{cc} L^{Q} & =\frac{3\omega_{Q}^{2}J_{0}^{Q}\left(0\right)+6\omega_{1}^{2}\left(J_{0}^{Q}\left(0\right)+J_{0}^{Q}\left(\lambda_{1}\right)\right)}{2\lambda_{1}^{2}}+\frac{5J_{1}^{Q}\left(\omega_{0}\right)}{2}+2J_{2}^{Q}\left(2\omega_{0}\right)\;\mathrm{and}\;\\ L^{D} & =\frac{2\left(\lambda_{2}J_{0}^{D}\left(\lambda_{3}\right)+\lambda_{3}J_{0}^{D}\left(\lambda_{2}\right)\right)}{9\lambda_{1}}+\frac{J_{0}^{D}\left(\omega_{0}-\omega_{S}\right)}{18}+\frac{J_{1}^{D}\left(\omega_{0}\right)}{6}+\frac{J_{1}^{D}\left(\omega_{S}\right)}{3}+\frac{J_{2}^{D}\left(\omega_{0}+\omega_{S}\right)}{3} \end{array} \end{array}$$

(A26g) $$\begin{array}{c} \begin{array}{cc} M^{Q} & =\frac{3\omega_{Q}^{2}J_{0}^{Q}\left(0\right)+2\omega_{1}^{2}\left(J_{0}^{Q}\left(0\right)+J_{0}^{Q}\left(\lambda_{1}\right)\right)}{2\lambda_{1}^{2}}+\frac{J_{1}^{Q}\left(\omega_{0}\right)}{2}+J_{2}^{Q}\left(2\omega_{0}\right)\;\mathrm{and}\;\\ M^{D} & =\frac{2\left(\lambda_{2}J_{0}^{D}\left(\lambda_{3}\right)+\lambda_{3}J_{0}^{D}\left(\lambda_{2}\right)\right)}{9\lambda_{1}}+\frac{5J_{0}^{D}\left(\omega_{0}-\omega_{S}\right)}{18}+\frac{5J_{1}^{D}\left(\omega_{0}\right)}{6}+\frac{J_{1}^{D}\left(\omega_{S}\right)}{3}+\frac{5J_{2}^{D}\left(\omega_{0}+\omega_{S}\right)}{3} \end{array} \end{array}$$

(A26h) $$\begin{array}{c} \begin{array}{cc} K^{Q} & =\frac{3\omega_{1}\omega_{Q}\left(J_{0}^{Q}\left(0\right)-J_{0}^{Q}\left(\lambda_{1}\right)\right)}{2\lambda_{1}^{2}}\;\mathrm{and}\;\\ K^{D} & =\frac{2\omega_{1}\left(J_{0}^{D}\left(\lambda_{3}\right)-J_{0}^{D}\left(\lambda_{2}\right)\right)}{9\lambda_{1}} \end{array} \end{array}$$

As mentioned above, the quadrupolar relaxation mechanism (implying the $T_{20}$ coherence) samples the spectral densities at the angular velocity $\lambda_{1}$, while the heteronuclear dipolar relaxation mechanism (implying the $T_{10}$ coherence) samples the two other angular velocities ($\lambda_{2}$ and $\lambda_{3}$), extending notably, the dynamical range probed by spin-locking relaxation measurements.
